# Comparing SGLT2i and Other Oral Antidiabetic Drugs as Dual Therapy Add‐On to Metformin in Type 2 Diabetes: A Systematic Review and Meta‐Analysis

**DOI:** 10.1002/edm2.70176

**Published:** 2026-02-12

**Authors:** Yuhang Ma, Yi Lin, Xiaoying Ding, Yongde Peng

**Affiliations:** ^1^ Department of Endocrinology and Metabolism, Shanghai General Hospital Shanghai Jiaotong University School of Medicine Shanghai China

**Keywords:** diabetes mellitus, hypoglycaemic agents, meta‐analysis, metformin, sodium‐glucose transporter 2 inhibitors, systematic review

## Abstract

**Aims:**

To compare up‐to‐date efficacy and safety data of sodium‐glucose cotransporter‐2 inhibitors (SGLT‐2is) versus other metformin‐containing oral dual‐therapies (ODTs) in type 2 diabetes mellitus.

**Methods:**

We updated a 2016 systematic literature review (SLR), searching MEDLINE, Embase, the Cochrane Database of Systematic Reviews, congress abstracts and SLR/meta‐analysis (MA) bibliographies to identify randomised controlled trials. Two independent reviewers determined eligible studies, which were extracted and assessed using the Cochrane Risk of Bias tool. MA was conducted using random‐effects pairwise models.

**Results:**

We included 36 publications (23 unique studies). Mean differences (MD) indicated comparable change in HbA_1c_ from baseline at Weeks 24 and 52 between SGLT‐2i plus metformin and other metformin‐containing ODTs. Patients on SGLT‐2i plus metformin showed a significantly lower risk of hypoglycaemia (risk ratio [RR]: 0.27, 95% CI: 0.09, 0.84) and significantly greater weight reduction (MD: –2.59; 95% CI: −4.42, −0.77), but an elevated risk of genital infection (RR: 5.08; 95% CI: 3.49, 7.38) at Week 52 compared with other ODTs. Other safety outcomes (e.g., urinary tract infections) were comparable between SGLT‐2i plus metformin and other ODTs.

**Conclusion:**

Compared with other ODTs, SGLT‐2i plus metformin exhibited overall comparable efficacy and safety, with a lower risk of hypoglycaemia and greater weight reduction at Week 52.

## Graphical abstract

This systematic literature review and meta‐analysis compared sodium‐glucose cotransporter‐2 inhibitors (SGLT‐2is) against other metformin‐containing oral dual‐therapies (ODTs) in type 2 diabetes mellitus. SGLT‐2i plus metformin exhibited overall comparable efficacy and safety, with a lower risk of hypoglycaemia and greater weight reduction than other ODTs at Week 52.

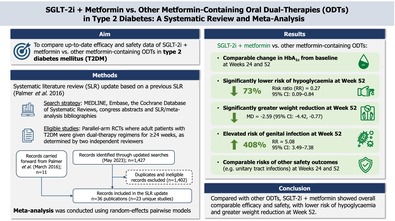

## 1 Introduction

Over 90% of diabetes cases are type 2 diabetes mellitus (T2DM), representing a major global health concern [1]. Diabetes affected approximately 589 million adults worldwide in 2024, a number projected to rise to 852.5 million by 2050 [1]. China contains the highest number of adults with diabetes (148.0 million in 2024) [1]. Effective management of T2DM is crucial to maintain optimal blood glucose levels, prevent or delay complications and improve patient outcomes [2, 3]. While lifestyle modifications are first‐line interventions for managing T2DM, many patients require pharmacological intervention to achieve glycaemic control [3, 4].

Metformin is a common treatment for T2DM management due to its efficacy and safety profile [5]. It works by lowering glucose production in the liver and improving insulin sensitivity in muscle and fat tissues [6]. The American Diabetes Association (ADA) guidelines include metformin as part of high‐efficacy approaches to achieve and maintain glycaemic and weight management goals [7]. Among patients who do not achieve glycaemic targets with lifestyle modifications and/or monotherapy (e.g., metformin alone), dual‐therapy regimens are often prescribed [4].

Dual‐therapy regimens combine two classes of antidiabetic treatments with complementary mechanisms of action, which can lead to greater glycaemic control than either treatment alone [7]. Aside from separate administrations of individual products, dual fixed‐dose combination (FDC) products are also available [7]. The ADA guidelines note that stepwise addition of other drugs to metformin should not be delayed in patients who fail to reach glycaemic targets [7]. Additionally, evidence suggests that early combination treatment may lead to a slower decline in glycaemic control compared to metformin monotherapy [7]. Thus, initial combination treatment could be considered in some patients, such as those with glycated haemoglobin A1c (HbA_1c_) levels 1.5%–2.0% above their individualised goal [7]. Sodium‐glucose cotransporter‐2 inhibitors (SGLT‐2is) are a class of antidiabetic drugs that inhibit glucose reabsorption in the proximal tubule of the kidneys, reducing glucose reabsorption and increasing urinary glucose excretion independently of insulin [8, 9]. SGLT‐2is also exhibited favourable cardiorenal‐protective effects, including reduction in cardiovascular and all‐cause mortality, risk of chronic kidney disease progression and hospitalisation for heart failure [10, 11, 12, 13]. Both the American Association of Clinical Endocrinology (AACE) and ADA guidelines recommend SGLT‐2is or glucagon‐like peptide‐1 receptor agonists (GLP‐1RAs) in patients with atherosclerotic cardiovascular disease, heart failure or chronic kidney disease [7, 14].

As dual‐therapy regimens such as metformin and SGLT‐2i are widely used in clinical settings, it is important to understand the current comparative treatment landscape to inform clinicians' decision‐making between combination treatment options for different patient populations. A systematic literature review (SLR) and meta‐analysis (MA) by Palmer et al. in 2016 analysed 301 randomised controlled trials (RCTs) evaluating clinical outcomes and adverse events (AEs) of glucose‐lowering drugs in patients with T2DM [15]. Palmer et al. 2016 concluded that there were no significant differences in the odds of all‐cause mortality between drug classes, whether as monotherapy or metformin‐containing combination regimens [15]. There were mixed results for differences among metformin dual‐therapy regimens for other outcomes; for example, compared to metformin plus sulfonylurea or dipeptidyl peptidase‐4 inhibitors (DPP‐4i), SGLT‐2i plus metformin was associated with increased odds of stroke, but lower odds of treatment failure and hypoglycaemia, as well as significantly lower body weight [15].

Since Palmer et al. was conducted in 2016, new dual‐therapy regimens have become available, alongside new evidence and updates to guidelines in diabetes [7]. For instance, recent studies indicate that SGLT‐2i as adjunct therapy to metformin could lead to improved efficacy and safety compared with other oral antidiabetic drugs (OADs) with metformin, for some outcomes such as HbA_1c_ reduction and weight loss [16, 17].

The objective of this study was thus to identify up‐to‐date data to compare the efficacy and safety of dual therapy with SGLT‐2is versus other OADs as add‐ons to metformin in the treatment of T2DM to inform clinicians' decision‐making between combination treatment options.

## 2 Methods

The SLR update used a pre‐specified protocol based on the SLR methodology in Palmer et al. 2016 [15], with the addition of Cochrane Database of Systematic Reviews (CDSR) and congress searches. This protocol was not prospectively registered. The SLR and MA were performed by following recommendations from the Cochrane Collaboration and guidelines from the University of York Centre for Reviews and Dissemination (CRD), and are reported in accordance with the preferred reporting items for systematic reviews and meta‐analyses (PRISMA) statement [18, 19, 20]. MEDLINE, Embase, the Cochrane Central Register of Controlled Trials (CENTRAL) and CDSR databases were searched on 24th May 2023 via the Ovid SP platform for studies published since 2016. Abstracts from the ADA and European Association for the Study of Diabetes (EASD) congresses from 2021 to 2023 were also searched; high‐quality studies published as congress abstracts before 2021 were assumed to have since been published as peer‐reviewed journal articles. Additionally, the bibliographies of Palmer et al. 2016 as well as any SLRs and (network) MAs ([N]MAs) identified in the database searches and which met the eligibility criteria at the abstract review stage were hand‐searched to identify studies relevant to this update. The search strategy is presented in Table S1.

The eligibility criteria are presented in Table S2. Briefly, studies were eligible if they were parallel‐arm RCTs in which adult patients with T2DM were given dual‐therapy regimens (metformin plus SGLT‐2i vs. any other oral metformin‐containing dual‐therapy regimens) for ≥ 24 weeks, and reported relevant efficacy and safety outcomes. Abstracts and full‐texts were reviewed by two independent reviewers, with disagreements resolved through discussion to reach consensus. If necessary, a third reviewer made the final decision. Publications reporting on the same study were linked and considered as one unit.

Key information from eligible studies was extracted by one individual into a prespecified data extraction grid. A second individual subsequently verified the extracted information and checked that no relevant information had been missed. Data for eligible studies presented in Palmer et al. 2016 were transferred to the data extraction grid; as individual study outcomes were not reported in Palmer et al. 2016, these were extracted from each study publication [15]. Each study was assessed using the Cochrane Risk of Bias tool [21].

A detailed assessment of study design, patient characteristics, interventions and outcome definitions/timepoints was conducted to ensure the selected studies were sufficiently similar for a MA. Patient populations across the studies were largely similar and hence were considered comparable in terms of baseline characteristics (Table S3). Week 24, Week 26 and Month 6 timepoints reported across studies were considered to be sufficiently close, and were taken to be the same ‘Week 24’ timepoint. The main analyses compared outcomes for metformin with SGLT‐2i against other dual therapies of interest (as a composite group): metformin with DPP‐4i, sulfonylureas (SU) or GLP‐1RA. Subgroup analyses were conducted for SGLT‐2i plus metformin versus DPP‐4i plus metformin and SGLT‐2i plus metformin versus SU plus metformin. As there was only one GLP‐1RA study included, a subgroup analysis for GLP‐1RA plus metformin was not possible.

Analyses were conducted of outcomes at Week 24 and/or Week 52. Efficacy outcomes of interest were: HbA_1c_ change from baseline; HbA_1c_ level at follow‐up; treatment failure and weight change from baseline. Safety outcomes of interest were: hypoglycaemia; diarrhoea; urinary tract infection (UTI); genital infection; stroke; all‐cause mortality; myocardial infarction (MI); overall serious AEs.

In alignment with Palmer et al. 2016, random‐effects pairwise models were employed in the analyses [15]. Between‐study variance used in the random effects was estimated using the DerSimonian and Laird procedure [22]. A continuity correction of 0.5 was applied in studies with zero cell frequencies. Estimated treatment effects for continuous and binary outcomes were mean differences (MD) and risk ratios (RR), respectively, with pooled estimates obtained using the inverse variance method [23]. Corresponding confidence intervals (CIs) were reported for all estimates. Heterogeneity was reported using the I^2^ statistic. Funnel plots were used to visualise any potential biases exhibited by studies. All analyses were conducted in R (v4.3.0 onwards) [24].

## 3 Results

**FIGURE 1:**
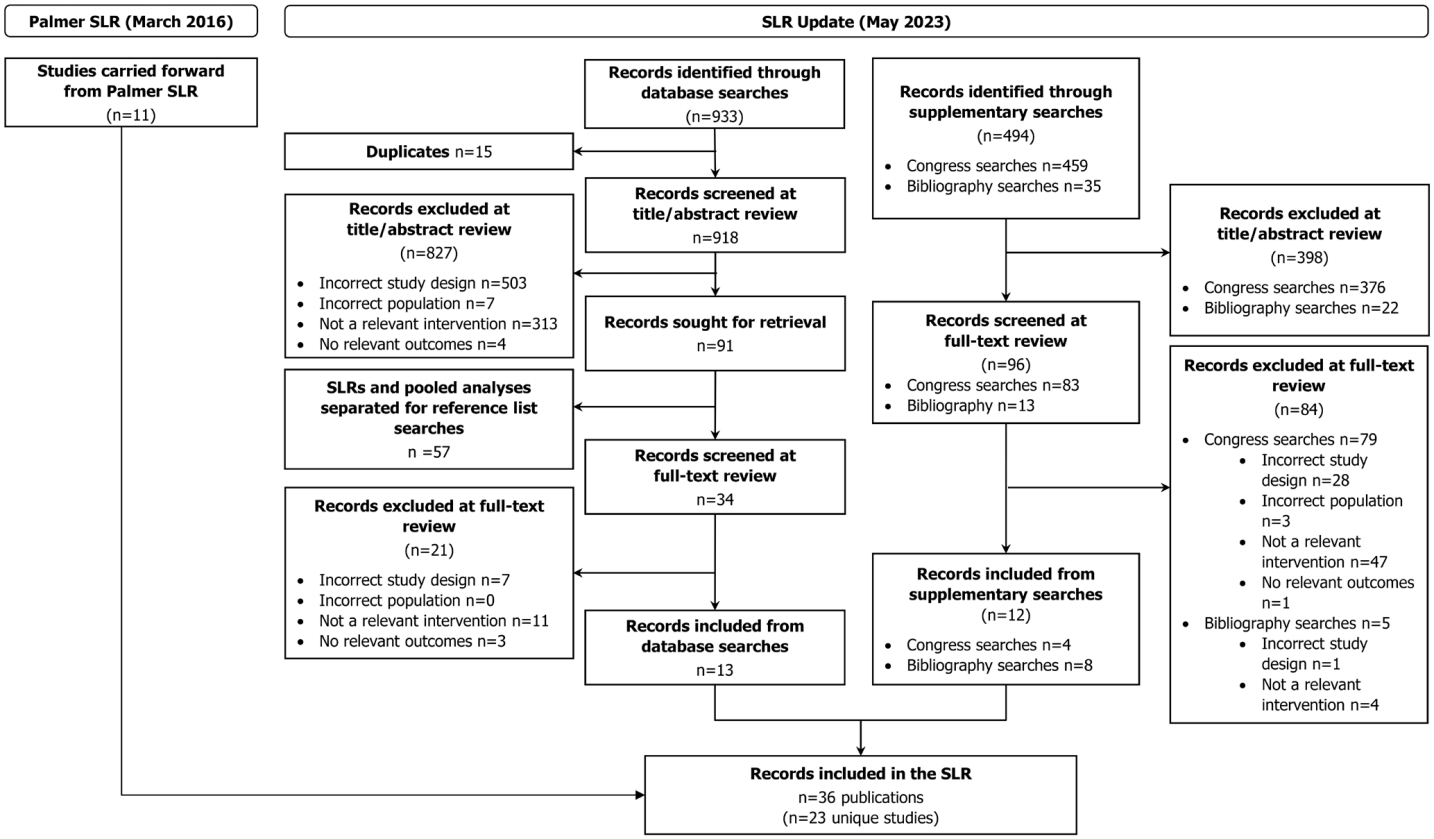
PRISMA flow diagram for the SLR. Abbreviations: SLR, systematic literature review.

The Preferred Reporting Items for Systematic Reviews and Meta‐Analyses (PRISMA) flow diagram for the SLR is presented in Figure 1. A total of 918 records were reviewed from the electronic databases, with 13 records ultimately included. From the supplementary searches, 494 records were reviewed and 12 were included. Finally, 11 studies from the original SLR (Palmer et al. 2016) were relevant to the current scope and carried forward for data extraction [15]. Ultimately, 36 publications reporting on 23 unique studies were included and extracted (Table 1). All 23 studies were deemed similar enough for the MA.

**TABLE 1:** Characteristics of studies included in the SLR.

#	NCT number (study name)	Author (year)	Interventions (*N* patients per treatment group)	Outcomes reported (efficacy)	Outcomes reported (safety)
1	**NCT01167881 (EMPA‐REG H2H‐SU)**	**Ridderstråle (2014)** [25]	Glimepiride (*n* = 780) Empagliflozin (*n* = 765)	HbA_1c_ levels Change in HbA_1c_ levels from baseline Treatment failure	Cardiovascular mortality All‐cause mortality MI Stroke Heart failure Death from renal or cardiovascular causes Overall serious AEs Hypoglycaemia GI AE UTI Genital infection
2		Chirila (2016) [26]			
3		Ridderstrale (2018) [27]			
4	**NCT03851432**	**Gao (2023)** [28]	Janagliflozin (*n* = 139) Placebo (*n* = 132)	Change in HbA_1c_ levels from baseline Treatment failure	All‐cause mortality Overall serious AEs Hypoglycaemia Change in body weight from baseline UTI Genital infection
5	**NCT02769481**	**Halvorsen (2023)** [29]	Bexagliflozin (*n* = 213) Glimepiride (*n* = 213)	HbA_1c_ levels Change in HbA_1c_ levels from baseline Treatment failure	All‐cause mortality Overall serious AEs Hypoglycaemia Body weight Change in body weight from baseline GI AE UTI Genital infection
6	**NCT01159600 (EMPA‐REG MET)**	**Häring (2014)** [30]	Empagliflozin 10 mg (*n* = 217) Empagliflozin 25 mg (*n* = 213) Placebo (*n* = 207)	HbA_1c_ levels Change in HbA_1c_ levels from baseline	All‐cause mortality Overall serious AEs Hypoglycaemia Body weight Change in body weight from baseline UTI Genital infection
7		Inzucchi (2021) [31]			
8	**NR**	**Ishtiaque (2022)** [32]	Dapagliflozin (*n* = 90) Sitagliptin (*n* = 90)	HbA_1c_ levels	NR
9	**NCT05359432**	**Khan (2022)** [33]	Empagliflozin (*n* = 53) Vildagliptin (*n* = 54)	HbA_1c_ levels Change in HbA_1c_ levels from baseline	Overall serious AEs Body weight Change in body weight from baseline UTI
10	**N‐ISM**	**Kitazawa (2021a)** [34]	Ipragliflozin (*n* = 54) Sitagliptin (*n* = 57)	HbA_1c_ levels Change in HbA_1c_ levels from baseline	All‐cause mortality Overall serious AEs Hypoglycaemia Body weight Change in body weight from baseline GI AE UTI Genital infection
11		Kitazawa (2021b) [35]			
12	**NCT01505426**	**Lu (2016)** [36]	Ipragliflozin (*n* = 87) Placebo (*n* = 83)	Change in HbA_1c_ levels from baseline	Overall serious AEs Hypoglycaemia Change in body weight from baseline GI AE UTI Genital infection
13		Min (2017) [37]			
14	**NCT02863328 (PIONEER 2)**	**Rodbard (2019)** [38]	Semaglutide (oral; *n* = 411) Empagliflozin (*n* = 410)	Change in HbA_1c_ levels from baseline Treatment failure	All‐cause mortality MI Stroke Heart failure Overall serious AEs Hypoglycaemia Change in body weight from baseline GI AE UTI Genital infection
15		Montanya (2021) [39]			
16		Shabeer (2022) [40]			
17		Knop (2021) [41]			
18		Aroda (2022) [42]			
19		Rosenstock (2021) [43]			
20	**Tang 2019**	**Tang (2019)** [44]	Dapagliflozin (*n* = 18) Glimepiride (*n* = 18)	HbA_1c_ levels	NR
21	**NCT02630706 (VERTIS Asia)**	**Ji (2019)** [45]	Ertugliflozin 5 mg (*n* = 170) Ertugliflozin 15 mg (*n* = 169) Placebo (*n* = 167)	HbA_1c_ levels Change in HbA_1c_ levels from baseline Treatment failure	All‐cause mortality Overall serious AEs Hypoglycaemia Change in body weight from baseline UTI Genital infection
22	**NCT00968812 (CANTATA‐SU)**	**Cefalu (2013)** [46]	Glimepiride (*n* = 482) Canagliflozin 100 mg (*n* = 483) Canagliflozin 300 mg (*n* = 485)	Change in HbA_1c_ levels from baseline Treatment failure	Cardiovascular mortality All‐cause mortality MI Stroke Sustained decline in eGFR End‐stage kidney disease Overall serious AEs Hypoglycaemia Change in body weight from baseline UTI Genital infection
23		Leiter (2016) [47]			
24		Patel (2016) [48]			
25		Heerspink (2017) [49]			
26	**NCT01809327**	**Rosenstock (2016)** [50]	Canagliflozin 100 mg (*n* = 237) Canagliflozin 300 mg (*n* = 237) Placebo (*n* = 237)	HbA_1c_ levels Change in HbA_1c_ levels from baseline	Cardiovascular mortality All‐cause mortality Stroke Death from renal or cardiovascular causes Overall serious AEs Hypoglycaemia Change in body weight from baseline GI AE UTI Genital infection
27	**NCT02794792 (IMPRESSION)**	**Shestakova (2018)** [51]	Ipragliflozin 50 mg (*n* = 38) Ipragliflozin 100 mg (*n* = 69) Placebo (*n* = 14)	HbA_1c_ levels	Body weight
28	**NCT01095666**	**Yang (2016)** [52]	Dapagliflozin 5 mg (*n* = 147) Dapagliflozin 10 mg (*n* = 152) Placebo (*n* = 145)	Change in HbA_1c_ levels from baseline Treatment failure	All‐cause mortality Overall serious AEs Hypoglycaemia Change in body weight from baseline GI AE UTI Genital infection
29	**NCT00643851, NCT00859898**	**Henry (2012)** [53]	Dapagliflozin 5 mg (*n* = 194) Dapagliflozin 10 mg (*n* = 211) Placebo (Study 1, *n* = 201; Study 2, *n* = 208)	HbA_1c_ levels Change in HbA_1c_ levels from baseline Treatment failure	Cardiovascular mortality All‐cause mortality MI Death from renal or cardiovascular causes Overall serious AEs Hypoglycaemia Body weight Change in body weight from baseline GI AE UTI Genital infection
30	**NCT01106677 (CANTATA‐D)**	**Lavalle‐González (2013)** [54]	Canagliflozin 100 mg (*n* = 368) Canagliflozin 300 mg (*n* = 367) Sitagliptin (*n* = 366) Placebo (*n* = 183)	Change in HbA_1c_ levels from baseline Treatment failure	MI Stroke Overall serious AEs Hypoglycaemia Change in body weight from baseline GI AE UTI Genital infection
31	**NCT00528879**	**Bailey (2010)** [55]	Dapagliflozin 2.5 mg (*n* = 137) Dapagliflozin 5 mg (*n* = 137) Dapagliflozin 10 mg (*n* = 135) Placebo (*n* = 137)	HbA_1c_ levels Change in HbA_1c_ levels from baseline Treatment failure	All‐cause mortality Overall serious AEs Hypoglycaemia Body weight Change in body weight from baseline GI AE UTI Genital infection
32	**NCT00855166**	**Bolinder (2012)** [56]	Dapagliflozin (*n* = 89) Placebo (*n* = 91)	Change in HbA_1c_ levels from baseline Treatment failure	All‐cause mortality Overall serious AEs Hypoglycaemia Change in body weight from baseline GI AE UTI Genital infection
33	**NCT01135433 (ILLUMINATE)**	**Kashiwagi (2015)** [57]	Ipragliflozin (*n* = 112) Placebo (*n* = 56)	HbA_1c_ levels Change in HbA_1c_ levels from baseline	Overall serious AEs Hypoglycaemia Body weight Change in body weight from baseline GI AE
34	**NCT00660907**	**Nauck (2011)** [58]	Dapagliflozin (*n* = 406) Glipizide (*n* = 408)	Change in HbA_1c_ levels from baseline	All‐cause mortality MI Heart failure Overall serious AEs Hypoglycaemia Change in body weight from baseline GI AE UTI Genital infection
35	**NCT01422876**	**DeFronzo (2015)** [59]	Linagliptin (*n* = 128) Empagliflozin 25 mg (*n* = 140) Empagliflozin 10 mg (*n* = 137)	Change in HbA_1c_ levels from baseline	All‐cause mortality MI Overall serious AEs Change in body weight from baseline GI AE UTI
36	**NR**	**Efstathiou (2015)** [60]	Saxagliptin (*n* = 34) Dapagliflozin (*n* = 32)	Change in HbA_1c_ levels from baseline	Hypoglycaemia Change in body weight from baseline

*Note:* **Bolded** studies were used as the primary publication for extractions.

Abbreviations: AE, adverse event; GI, gastrointestinal; HbA_1c_, glycated haemoglobin A1c; MI, myocardial infarction; NCT, National Clinical Trial; NR, not reported; SLR, systematic literature review; UTI, urinary tract infection.

Results of the Risk of Bias assessment are available in Table S4. Among the included studies, none were assessed to be at high risk of selection bias; 19 (82.6%) trials had low risk of bias in sequence generation (4 unclear), but 13 (56.5%) had unclear risk of allocation concealment (10 assessed as low‐risk). Risk of performance bias in the masking of participants/investigators was low in most trials (17; 73.9%) but high/unclear in six trials (26.1%). Risk of detection bias in outcome assessment was high/unclear in 20 studies (87.0%). High/unclear risk of attrition bias was observed in eight studies (34.8%), while only one study (4.4%) had a high risk of selective outcome reporting. The trial sponsor was involved in authorship and/or data management for 18 studies (78.3%). Funnel plots illustrating the potential biases exhibited by studies are presented in Figure S1,S2.

### 3.1 Efficacy Analysis

**FIGURE 2:**
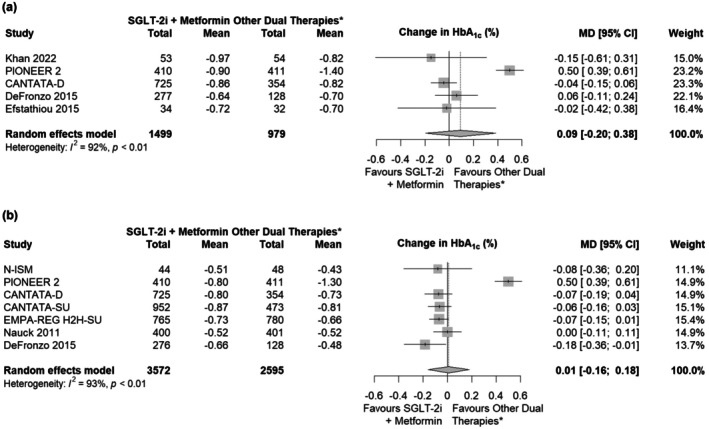
Results from efficacy analyses of SGLT‐2i plus metformin for change in HbA_1c_ at (a) Week 24 in main analysis and (b) Week 52 in main analysis. *Consisting of DPP‐4i, GLP‐1RA, or SU in combination with metformin. Abbreviations: CI, confidence interval; DPP‐4i, dipeptidyl peptidase‐4 inhibitors; GLP‐1RA, glucagon‐like peptide‐1 receptor agonist; HbA_1c_, glycated haemoglobin A1c; MD, mean difference; RR, relative risk; SGLT‐2i, sodium‐glucose cotransporter‐2 inhibitors; SU, sulphonylurea.

In the analysis of change in HbA_1c_ from baseline, the MDs were comparable between patients receiving SGLT‐2i plus metformin and other metformin‐containing dual therapies at Week 24 (0.09; 95% CI: −0.20, 0.38; Figure 2a) and Week 52 (0.01; 95% CI: −0.16, 0.18; Figure 2b). Heterogeneity between studies was high (I^2^ = 92% for Week 24; I^2^ = 93% for Week 52). For the subgroup analysis comparing SGLT‐2i plus metformin against SU plus metformin, insufficient data were available for a Week 24 analysis, but at Week 52, there was no significant difference for change in HbA_1c_ (MD: –0.05; 95% CI: −0.11, 0.00; Figure S3a). In the subgroup analyses comparing SGLT‐2i plus metformin against DPP‐4i plus metformin, the MD was not statistically significant at Week 24 (MD: –0.02; 95% CI: −0.11, 0.07; Figure S3b), but by Week 52, patients on SGLT‐2i had a significantly larger reduction in HbA_1c_ (MD –0.11; 95% CI: −0.19, −0.02; Figure S3c) compared with patients on DPP‐4i plus metformin.

For the analysis of HbA_1c_ level at Week 24, patients on SGLT‐2i plus metformin had significantly higher levels of HbA_1c_ than patients on other dual therapies (MD: 0.19; 95% CI: 0.07, 0.32; Figure S3d); heterogeneity between the studies was low (I^2^ = 0%). In the subgroup analysis comparing against DPP‐4i plus metformin, patients on SGLT‐2i plus metformin also had significantly higher levels of HbA_1c_ at Week 24 (MD: 0.20; 95% CI: 0.07, 0.33; Figure S3e).

**FIGURE 3:**
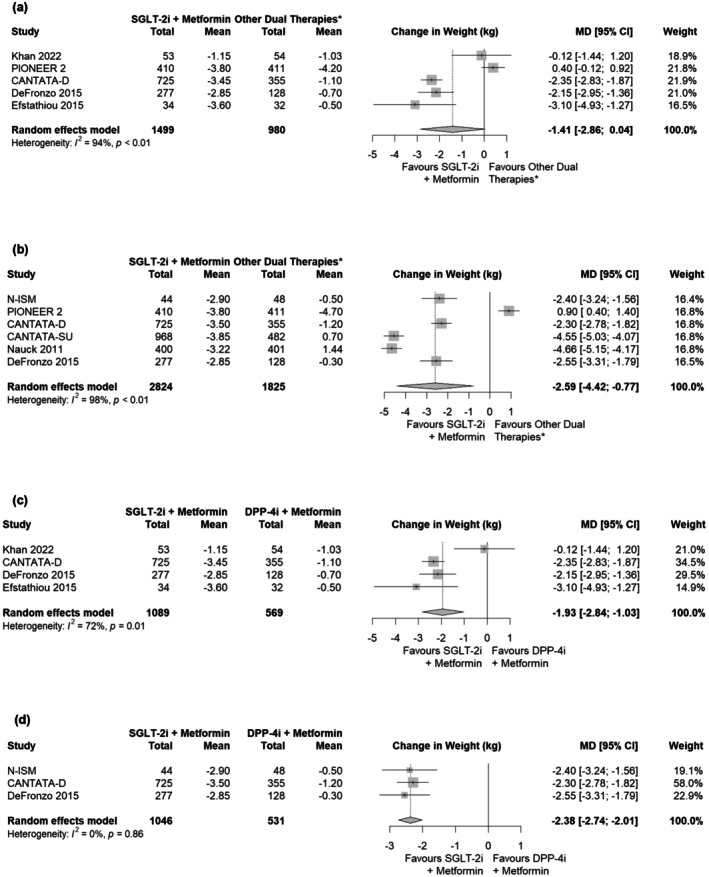
Results from safety analysis of SGLT‐2i plus metformin for change in weight from baseline at (a) Week 24 in main analysis, (b) Week 52 in main analysis, (c) Week 24 for subgroup analysis comparing against DPP‐4i plus metformin and (d) Week 52 for subgroup analysis comparing against DPP‐4i plus metformin. *Consisting of DPP‐4i, GLP‐1RA, or SU in combination with metformin. Abbreviations: CI, confidence interval; DPP‐4i, dipeptidyl peptidase‐4 inhibitors; GLP‐1RA, glucagon‐like peptide‐1 receptor agonist; kg, kilogram; MD, mean difference; SGLT‐2i, sodium‐glucose cotransporter‐2 inhibitors; SU, sulphonylurea.

In the analysis of mean change in weight from baseline, there was no significant difference at Week 24 between patients receiving SGLT‐2i plus metformin versus other dual therapies (MD: –1.41; 95% CI: −2.86, 0.04; Figure 3a). However, by Week 52, patients on SGLT‐2i plus metformin had significantly greater reductions in weight than patients on other dual therapies (MD: –2.59; 95% CI: −4.42, −0.77; Figure 3b). Subgroup analyses for change in weight were only possible for the DPP‐4i plus metformin subgroup; patients on SGLT‐2i plus metformin had significantly greater reductions in weight at both Weeks 24 (MD: –1.93; 95% CI: −2.84, −1.03; Figure 3c) and 52 (MD: –2.38; 95% CI: −2.74, −2.01; Figure 3d) than patients on DPP‐4i plus metformin.

There was no significant difference in risk of treatment failure at Week 52 between patients on SGLT‐2i plus metformin and other dual therapies (RR: 0.72; 95% CI: 0.49, 1.04; Figure S3f); heterogeneity between studies was high (I^2^ = 79%).

### 3.2 Safety Analysis

**FIGURE 4:**
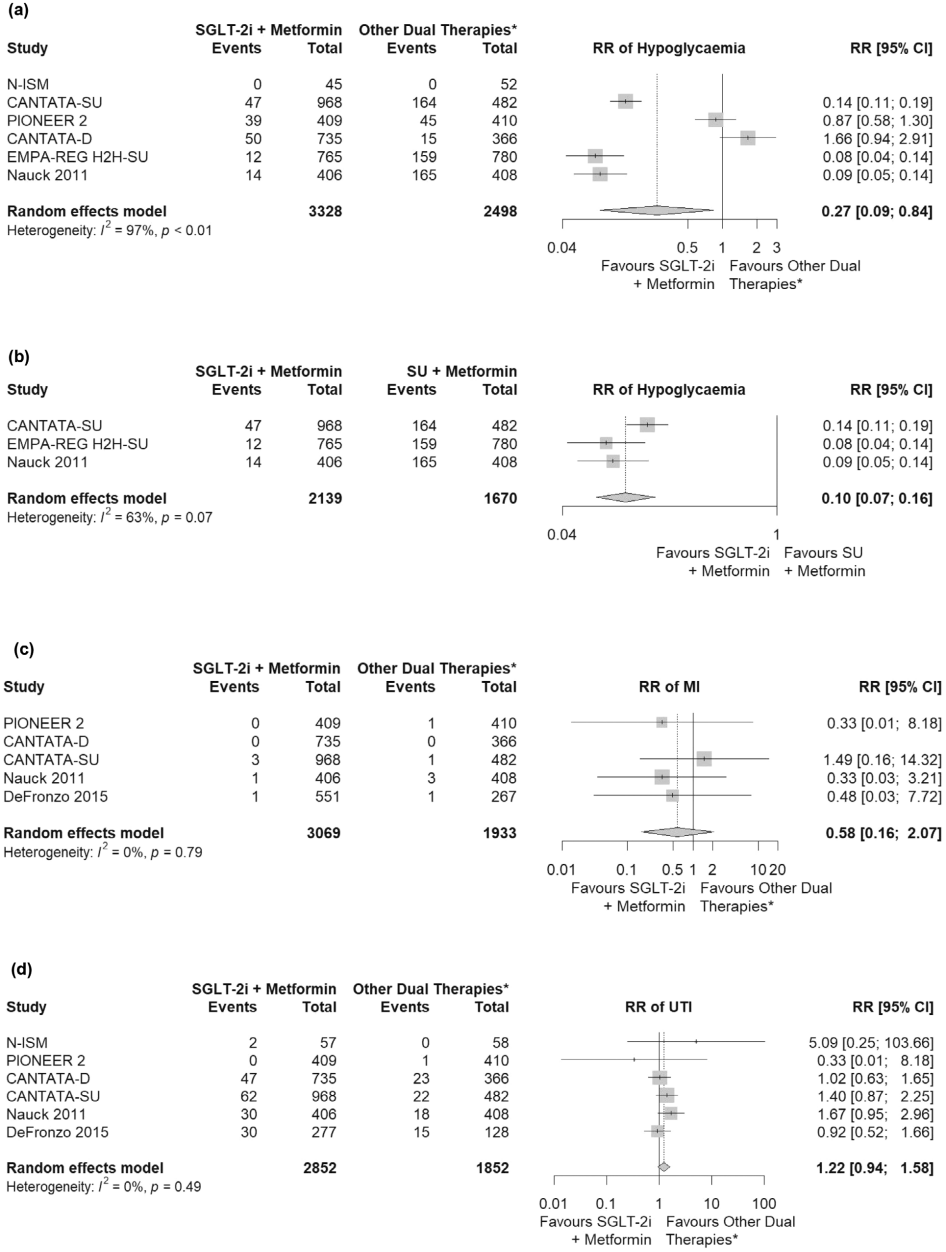
Results from safety analysis of SGLT‐2i plus metformin at Week 52 for (a) hypoglycaemia in main analysis, (b) hypoglycaemia in subgroup analysis against SU plus metformin, (c) MI in main analysis and (d) UTI in main analysis. *Consisting of DPP‐4i, GLP‐1RA, or SU in combination with metformin. Abbreviations: CI, confidence interval; DPP‐4i, dipeptidyl peptidase‐4 inhibitors; GLP‐1RA, glucagon‐like peptide‐1 receptor agonist; MI, myocardial infarction; RR, relative risk; SGLT‐2i, sodium‐glucose cotransporter‐2 inhibitors; SU, sulphonylurea; UTI, urinary tract infection.

Patients receiving SGLT‐2i plus metformin had a significantly lower risk of hypoglycaemia than patients receiving other metformin‐containing dual therapies at Week 52 (RR: 0.27; 95% CI: 0.09, 0.84; Figure 4a). There was high heterogeneity between the studies included in this analysis (I^2^ = 97%); one study (N‐ISM) was excluded from the estimate due to no observed events in either arm. In the subgroup analysis comparing against SU plus metformin, patients on SGLT‐2i plus metformin had a significant 90% lower risk of hypoglycaemia at Week 52 (RR: 0.10; 95% CI: 0.07, 0.16; Figure 4b).

The risk of MI was lower in patients receiving SGLT‐2i plus metformin than those receiving other metformin‐containing dual therapies at Week 52; however, the difference was not statistically significant (RR: 0.58; 95% CI: 0.16, 2.07; Figure 4c). Patients on SGLT‐2i plus metformin had an elevated but statistically comparable risk of UTI at Week 52 with patients receiving other dual therapies (RR: 1.22; 95% CI: 0.94, 1.58; Figure 4d). In the subgroup analysis comparing SGLT‐2i plus metformin against DPP‐4i plus metformin, the risk of UTI at Week 52 was comparable (RR: 1.00; 95% CI: 0.69, 1.45; Figure S4a). However, for the risk of genital infection, patients on SGLT‐2i plus metformin had five times higher risk compared to other dual therapies at Week 52 (RR: 5.08; 95% CI: 3.49, 7.38; Figure S4b).

The risk of overall serious AEs at Week 52 was similar among therapy groups in the main analysis (RR: 0.83; 95% CI: 0.65, 1.08; Figure S5a) and in the subgroup analysis comparing DPP‐4i plus metformin against SGLT‐2i plus metformin (RR: 0.79; 95% CI: 0.50, 1.25; Figure S5b). Likewise, there was no significant difference in the risk of all‐cause mortality (RR: 0.63; 95% CI: 0.17, 2.37), stroke (RR: 3.34; 95% CI: 0.58, 19.37) or diarrhoea (RR: 0.68; 95% CI: 0.36, 1.26) between patients receiving SGLT‐2i plus metformin versus other dual therapies at Week 52 (Figure S6).

## 4 Discussion

### 4.1 Efficacy

This SLR and MA provide the most up‐to‐date synthesis of evidence comparing SGLT2 inhibitors with other metformin‐based dual therapies in T2DM, including more recent published data on combinations with newer agents like GLP‐1RAs. Results demonstrated that SGLT‐2i plus metformin was as effective as other metformin‐containing dual therapies, consistent with the recommendation of its use in international guidelines [7, 14].

The MA results revealed that SGLT‐2i plus metformin was as effective as the composite comparator consisting of metformin plus DPP‐4i, GLP‐1RA or SU when looking at the change in HbA_1c_. A previous 2017 MA found SGLT2 inhibitors plus metformin treatment to be associated with significantly greater change in HbA_1c_ from baseline versus non‐SGLT2 combinations at 1 year [61]; however, in this MA, the comparator metformin combinations included only DPP‐4i and SU.

In the subgroup analyses, patients on SGLT‐2i plus metformin had a significantly larger reduction in HbA_1c_ than those on DPP‐4i plus metformin at Week 52. A previous SLR and MA from 2017 similarly found that SGLT‐2i plus metformin was associated with a greater reduction in HbA_1c_ from baseline, compared with non‐SGLT‐2i metformin combinations [61]. This difference in efficacy also aligns with trends observed in real‐world analyses, including a population‐based study conducted in Italy [62], as well as a target trial emulation using a large, representative routine health dataset from the UK, which found that SGLT‐2i as adjunct therapy to metformin was more effective than DPP‐4i or SU in reducing mean HbA_1c_ levels at 1 year [16]. This result may be due to differences in the mechanism of action, as SGLT‐2i directly reduces serum glucose by inhibiting reabsorption in the kidney, whereas DPP‐4i's glucose‐lowering effect is indirect, increasing insulin and reducing glucagon release through hormone‐related pathways [63, 64].

PIONEER 2 was the only RCT comparing SGLT‐2i plus metformin and oral GLP‐1RA plus metformin [38]. In PIONEER 2, patients on oral GLP‐1RA plus metformin had significantly greater reduction in HbA_1c_ compared with those on SGLT‐2i plus metformin at Weeks 24 and 52 [38]. However, the use of GLP‐1RA has been reported to increase the risk of serious AEs and discontinuation due to AEs, compared with SGLT‐2is [65]. In PIONEER 2, more patients discontinued treatment due to AEs with GLP‐1RA than SGLT‐2i, largely driven by gastrointestinal disorders (8.0% vs. 0.7%) [38]. As no other study included in this SLR reported on these comparators, no comparative pooled estimates on efficacy or safety outcomes could be derived between SGLT‐2i plus metformin and GLP‐1RA plus metformin in the MA.

Compared to other examined dual therapies, patients on SGLT‐2i plus metformin had a substantial reduction in weight at Week 52. Weight loss with SGLT‐2is may be due to an initial diuretic effect, as well as excretion of glucose through the urine [55, 66]. Metformin may also induce weight loss by suppressing appetite through a range of mechanisms including: lactate‐mediated acidosis, increased secretion of appetite‐suppressing neuropeptides such as GLP‐1 and reduced bile acid absorption [67, 68]. A previous review found that SGLT‐2i plus metformin was significantly more effective in weight reduction than metformin monotherapy in patients with T2DM [69]. Therefore, SGLT‐2i plus metformin may be a more effective weight reduction option than monotherapy. Among the individual studies included in our estimates, PIONEER 2 (comparing oral GLP‐1RA plus metformin) was the only study reporting a weight reduction benefit over SGLT‐2i plus metformin [38]. Another review of weight‐centric treatments in T2DM describes both GLP‐1RA and SGLT‐2i as medications promoting weight loss [70]. The subgroup analyses in this study support the benefits of SGLT‐2i plus metformin in terms of weight reduction, with significantly greater reductions than those on DPP‐4i plus metformin at Weeks 24 and 52. Indeed, DPP‐4i medications have previously been highlighted as likely ‘weight neutral’, while SGLT‐2i medications promote weight loss [70]. This finding is reflected in real‐world evidence studies indicating that SGLT‐2i plus metformin was associated with a greater reduction in body mass index (BMI) and body weight at 1 year from baseline, compared with SU or DPP‐4i plus metformin [16, 62].

### 4.2 Safety

This MA's results demonstrated that SLGT‐2i plus metformin was generally as safe as other examined dual therapies. SGLT‐2i treatment saw a significant reduction in risk of hypoglycaemia at Week 52, but a significantly higher risk of genital infection, though the risk of UTI was similar to other dual therapies. Apart from statistical significance, it is also important to consider the clinical significance of outcomes [71, 72]. A survey of healthcare professionals working with T2DM patients highlighted hypoglycaemia, mortality, macrovascular outcomes including MI and stroke and HbA_1c_ levels as critical in the management of T2DM [72].

Patients on SGLT‐2i plus metformin demonstrated a significant 73% reduction in risk of hypoglycaemia (95% CI: 0.09, 0.84) compared to the other examined dual therapies at Week 52; this surpasses the threshold of 50% deemed as the minimal clinically important difference (MCID) for hypoglycaemic events among clinicians [72]. In the subgroup analysis comparing against SU plus metformin, the risk of hypoglycaemia among patients on SGLT‐2i plus metformin was even lower (RR: 0.10; 95% CI: 0.07, 0.16). This aligns with real‐world studies conducted in the US highlighting a lower risk of hypoglycaemia with SGLT2i compared to SU in patients with T2DM [73, 74]. CANTATA‐D was the only study included in the MA that investigated DPP‐4i plus metformin and recorded hypoglycaemic events, finding a similar risk of hypoglycaemia on SGLT‐2i and DPP‐4i [54], which is also supported by recent real‐world evidence [73]. The risk of MI was numerically lower in patients receiving SGLT‐2i plus metformin compared with other dual therapies, underscoring the use of SGLT‐2i in patients with high cardiorenal risk, in line with guideline recommendations [7, 14].

UTI is a well‐recognised AE of SGLT‐2i [7, 75]. While this analysis showed a numerically higher risk of UTI associated with SGLT‐2i plus metformin, the risk difference was not significant against the other examined dual therapies. Genital infection is also a commonly reported AE of SGLT‐2i, aligning with the findings in this study of increased risk associated with SGLT‐2i plus metformin compared to other dual therapies [75]. A UK nationwide claims database study also found a significantly higher risk of genital infection among patients with T2DM on SGLT‐2i compared to DPP‐4i [76]. However, a recently published consensus notes that most cases of genitourinary infection are of mild‐to‐moderate severity and can be managed with standard antimicrobial treatment without SGLT‐2i discontinuation [77].

### 4.3 Assessment of Bias

As many of the MAs in the current analysis included a small number of studies, statistical tests for funnel plot asymmetry may have limited power, and thus a visual assessment was conducted as a reasonable method for evaluating bias in this context [19, 78]. While the funnel plots should be interpreted with caution, there were no clear indications of potential biases in the majority of the outcomes analysed, as most studies fell within the funnel plots. The only exception was in the MA of hypoglycaemia at Week 52, where all studies fell outside of the funnel plot; this might suggest publication bias towards studies with certain characteristics or results, or against studies with nonsignificant or unfavourable hypoglycaemia results at Week 52.

PIONEER 2 was the only study that fell notably far from the funnel plot in multiple MAs. As described above, this was the only study that compared patients on GLP‐1RA plus metformin to those on SGLT‐2i plus metformin. The inclusion of PIONEER 2 in the MA of change in HbA_1c_ at Weeks 24 and 52 likely contributed to the high heterogeneity in the main analyses. In the quality assessment of PIONEER 2, there was a high risk of bias in both the performance (masking of participants/investigators) and detection (outcome assessment) domains, which might have contributed to the observations mentioned above.

### 4.4 Limitations

While this study was based on the methodology of Palmer et al. 2016, important adaptations were made to align with specific analysis aims focusing on SGLT‐2is, for example, in aggregating the other dual therapies into a composite group for the main MA. This impacts the direct comparability of findings against those of Palmer et al. 2016. Analyses were limited by a few events for outcomes such as MI. Previous work has suggested that alternatives to inverse variance methods may be more appropriate in these cases [79]. Future work should apply these alternative approaches. As doses of the same intervention were merged in the MA, a dose–response relationship could not be established in this analysis. Additionally, the number of head‐to‐head oral metformin‐containing dual therapy trials was limited, with most key outcomes supported by four or five studies. This could have been a driver of high I^2^ statistics, indicating a considerable percentage of variability. The number of head‐to‐head trials between SGLT‐2i plus metformin and specific dual‐therapy classes to support subgroup analyses was similarly limited. In particular, a reduced number of feasible subgroup analyses between SU plus metformin and SGLT‐2i plus metformin was available, as the SU plus metformin studies identified contained fewer outcomes that were of interest in our MA. Finally, potential confounding variables not measured in the analyses may have affected the pooled estimates. Given that only RCTs were included, there may be differences between the study populations and patients in the real‐world setting, limiting the generalisability of findings.

## 5 Conclusion

Overall, SGLT‐2i plus metformin appeared to be as effective as the other oral dual therapies investigated, but showed enhanced efficacy when specifically compared to DPP‐4i plus metformin and SU plus metformin, including greater reductions in weight at Week 52. SGLT‐2i plus metformin appeared to be as safe as the other examined dual therapies, except for the risk of genital infection. Patients on SGLT‐2i plus metformin exhibited a notably lower risk of hypoglycaemia than other oral dual therapies. Future research could investigate any potential efficacy and safety differences between co‐administered or FDC dual therapies, as well as triple therapies.

## Author Contributions

Yuhang Ma: Investigation, Writing – review and editing. Yi Lin: Formal analysis, Data curation, Writing – review and editing. Xiaoying Ding: Data curation, Writing – review and editing. Yongde Peng: Conceptualisation, Writing – review and editing. All authors have read and approved the final version of the manuscript for publication.

## Funding

The open‐access fee of this article was funded by AstraZeneca. AstraZeneca participated in study design; in the interpretation of data; in the writing of the report; and in the decision to submit the article for publication.

## Conflicts of Interest

The authors declare no conflicts of interest.

## Supporting information


**Table S1:** Search strategies.
**Table S2:** PICOS eligibility criteria for SLR update.
**Table S3:** Baseline patient characteristics.
**Table S4:** Risk of bias.
**Figure S1:** Funnel plots for main analyses.
**Figure S2:** Funnel plots for subgroup analyses comparing (a) DPP‐4i plus metformin and (b) SU plus metformin
**Figure S3:** Results from efficacy analyses of SGLT‐2i plus metformin for (a) change in HbA_1c_ at Week 52 in subgroup analysis against SU plus metformin, (b) change in HbA_1c_ at Week 24 in subgroup analysis against DPP‐4i plus metformin, (c) change in HbA_1c_ at Week 52 in subgroup analysis against DPP‐4i plus metformin, (d) HbA_1c_ level at Week 24 in main analysis, (e) HbA_1c_ level at Week 24 in subgroup analysis comparing against DPP‐4i plus metformin and (f) treatment failure at Week 52 in main analysis.
**Figure S4:** Results from safety analyses of SGLT‐2i plus metformin at Week 52 for (a) UTI in subgroup analysis comparing against DPP‐4i plus metformin and (b) genital infection in main analysis.
**Figure S5:** Results from safety analyses of SGLT‐2i plus metformin for overall serious AEs at Week 52 in (a) main analysis and (b) subgroup analysis comparing against DPP‐4i plus metformin.
**Figure S6:** Results from main safety analyses of SGLT‐2i plus metformin at Week 52 for (a) all‐cause mortality, (b) stroke and (c) diarrhoea.

## Data Availability

The data that support the findings of this study are available from the corresponding author upon reasonable request.

## References

[1] International Diabetes Federation , “IDF Diabetes Atlas 11th Edition 2025: Global Prevalence and Projections for 2050,” (2025), https://diabetesatlas.org/resources/idf‐diabetes‐atlas‐2025/. 10.1093/ndt/gfaf177 40874767

[2] C. Browning , A. Chapman , H. Yang , et al., “Management of Type 2 Diabetes in China: The Happy Life Club, a Pragmatic Cluster Randomised Controlled Trial Using Health Coaches,” BMJ Open 6, no. 3 (2016): e009319. 10.1136/bmjopen-2015-009319 PMC4785304 26944692

[3] M. Asif , “The Prevention and Control the Type‐2 Diabetes by Changing Lifestyle and Dietary Pattern,” Journal of Education Health Promotion 3 (2014): 1. 24741641 10.4103/2277-9531.127541 PMC3977406

[4] D. M. Nathan , J. B. Buse , M. B. Davidson , et al., “Medical Management of Hyperglycemia in Type 2 Diabetes: A Consensus Algorithm for the Initiation and Adjustment of Therapy: A Consensus Statement of the American Diabetes Association and the European Association for the Study of Diabetes,” Diabetes Care 32, no. 1 (2009): 193–203. 18945920 10.2337/dc08-9025 PMC2606813

[5] L. Schlender , Y. V. Martinez , C. Adeniji , et al., “Efficacy and Safety of Metformin in the Management of Type 2 Diabetes Mellitus in Older Adults: A Systematic Review for the Development of Recommendations to Reduce Potentially Inappropriate Prescribing,” BMC Geriatrics 17, no. 1 (2017): 227. 29047344 10.1186/s12877-017-0574-5 PMC5647555

[6] G. Rena , D. G. Hardie , and E. R. Pearson , “The Mechanisms of Action of Metformin,” Diabetologia 60, no. 9 (2017): 1577–1585. 28776086 10.1007/s00125-017-4342-z PMC5552828

[7] American Diabetes Association Professional Practice Committee , “Pharmacologic Approaches to Glycemic Treatment: Standards of Care in Diabetes—2024,” Diabetes Care 47 (2024): S158–S178. 38078590 10.2337/dc24-S009 PMC10725810

[8] E. Gronda , G. D. Lopaschuk , A. Arduini , et al., “Mechanisms of Action of SGLT2 Inhibitors and Their Beneficial Effects on the Cardiorenal Axis,” Canadian Journal of Physiology and Pharmacology 100, no. 2 (2022): 93–106. 35112597 10.1139/cjpp-2021-0399

[9] B. Rolek , M. Haber , M. Gajewska , S. Rogula , A. Pietrasik , and A. Gąsecka , “SGLT2 Inhibitors vs. GLP‐1 Agonists to Treat the Heart, the Kidneys and the Brain,” Journal of Cardiovascular Development and Disease 10, no. 8 (2023): 322. 37623335 10.3390/jcdd10080322 PMC10455499

[10] P. McEwan , P. D. Gabb , J. A. Davis , et al., “The Long‐Term Effects of Dapagliflozin in Chronic Kidney Disease: A Time‐To‐Event Analysis,” Nephrology, Dialysis, Transplantation 39, no. 12 (2024): 2040–2047. 10.1093/ndt/gfae106 PMC11596089 38730538

[11] C. Wanner , J. M. Lachin , S. E. Inzucchi , et al., “Empagliflozin and Clinical Outcomes in Patients With Type 2 Diabetes Mellitus, Established Cardiovascular Disease, and Chronic Kidney Disease,” Circulation 137, no. 2 (2018): 119–129. 28904068 10.1161/CIRCULATIONAHA.117.028268

[12] S. D. Wiviott , I. Raz , M. P. Bonaca , et al., “Dapagliflozin and Cardiovascular Outcomes in Type 2 Diabetes,” New England Journal of Medicine 380, no. 4 (2019): 347–357. 30415602 10.1056/NEJMoa1812389

[13] B. Zinman , C. Wanner , J. M. Lachin , et al., “Empagliflozin, Cardiovascular Outcomes, and Mortality in Type 2 Diabetes,” New England Journal of Medicine 373, no. 22 (2015): 2117–2128. 26378978 10.1056/NEJMoa1504720

[14] S. L. Samson , P. Vellanki , L. Blonde , et al., “American Association of Clinical Endocrinology Consensus Statement: Comprehensive Type 2 Diabetes Management Algorithm, 2023 Update,” Endocrine Practice 29, no. 5 (2023): 305–340. 37150579 10.1016/j.eprac.2023.02.001

[15] S. C. Palmer , D. Mavridis , A. Nicolucci , et al., “Comparison of Clinical Outcomes and Adverse Events Associated With Glucose‐Lowering Drugs in Patients With Type 2 Diabetes: A Meta‐Analysis,” Journal of the American Medical Association 316, no. 3 (2016): 313–324. 27434443 10.1001/jama.2016.9400

[16] P. Bidulka , D. G. Lugo‐Palacios , O. Carroll , et al., “Comparative Effectiveness of Second Line Oral Antidiabetic Treatments Among People With Type 2 Diabetes Mellitus: Emulation of a Target Trial Using Routinely Collected Health Data,” BMJ 385 (2024): e077097. 38719492 10.1136/bmj-2023-077097 PMC11077536

[17] B. Mishriky , R. Tanenberg , K. Sewell , and D. Cummings , “Comparing SGLT‐2 Inhibitors to DPP‐4 Inhibitors as an Add‐On Therapy to Metformin in Patients With Type 2 Diabetes: A Systematic Review and Meta‐Analysis,” Diabetes & Metabolism 44, no. 2 (2018): 112–120. 29477373 10.1016/j.diabet.2018.01.017

[18] Centre for Reviews and Dissemination , Systematic Reviews: CRD's Guidance for Undertaking Reviews in Health Care (CRD, 2008), https://www.york.ac.uk/media/crd/Systematic_Reviews.pdf.

[19] J. Higgins , J. Thomas , J. Chandler , et al., Cochrane Handbook for Systematic Reviews of Interventions (Cochrane, 2024), https://www.cochrane.org/authors/handbooks‐and‐manuals/handbook.

[20] M. J. Page , J. E. McKenzie , P. M. Bossuyt , et al., “The PRISMA 2020 Statement: An Updated Guideline for Reporting Systematic Reviews,” BMJ 372 (2021): n71. 33782057 10.1136/bmj.n71 PMC8005924

[21] J. P. Higgins , D. G. Altman , P. C. Gøtzsche , et al., “The Cochrane Collaboration's Tool for Assessing Risk of Bias in Randomised Trials,” BMJ (Clinical Research Ed) 343 (2011): d5928. 10.1136/bmj.d5928 PMC3196245 22008217

[22] R. DerSimonian and N. Laird , “Meta‐Analysis in Clinical Trials,” Controlled Clinical Trials 7, no. 3 (1986): 177–188. 3802833 10.1016/0197-2456(86)90046-2

[23] G. Schwarzer , J. Carpenter , and G. Rücker , Meta‐Analysis With R (Springer International, 2015), https://link.springer.com/book/10.1007/978‐3‐319‐21416‐0.

[24] “R Foundation for Statistical Computing,” (2023).

[25] M. Ridderstråle , K. R. Andersen , C. Zeller , G. Kim , H. J. Woerle , and U. C. Broedl , “Comparison of Empagliflozin and Glimepiride as Add‐On to Metformin in Patients With Type 2 Diabetes: A 104‐Week Randomised, Active‐Controlled, Double‐Blind, Phase 3 Trial,” Lancet Diabetes and Endocrinology 2, no. 9 (2014): 691–700. 24948511 10.1016/S2213-8587(14)70120-2

[26] C. Chirila , Q. Zheng , E. Davenport , et al., “Treatment Satisfaction in Type 2 Diabetes Patients Taking Empagliflozin Compared With Patients Taking Glimepiride,” Quality of Life Research 25, no. 5 (2016): 1199–1207. 26424170 10.1007/s11136-015-1140-2 PMC4840220

[27] M. Ridderstrale , J. Rosenstock , K. R. Andersen , H. J. Woerle , and A. Salsali , “Empagliflozin Compared With Glimepiride in Metformin‐Treated Patients With Type 2 Diabetes: 208‐Week Data From a Masked Randomized Controlled Trial,” Diabetes, Obesity & Metabolism 20, no. 12 (2018): 2768–2777. 10.1111/dom.13457 29961998

[28] L. Gao , Z. Cheng , B. Su , et al., “Efficacy and Safety of Janagliflozin as Add‐On Therapy to Metformin in Chinese Patients With Type 2 Diabetes Inadequately Controlled With Metformin Alone: A Multicentre, Randomized, Double‐Blind, Placebo‐Controlled, Phase 3 Trial,” Diabetes, Obesity & Metabolism 25, no. 3 (2023): 785–795. 10.1111/dom.14926 36433709

[29] Y. D. Halvorsen , J. P. Lock , J. P. Frias , et al., “A 96‐Week, Double‐Blind, Randomized Controlled Trial Comparing Bexagliflozin to Glimepiride as an Adjunct to Metformin for the Treatment of Type 2 Diabetes in Adults,” Diabetes, Obesity & Metabolism 25, no. 1 (2023): 293–301. 10.1111/dom.14875 36178197

[30] H. U. Häring , L. Merker , E. Seewaldt‐Becker , et al., “Empagliflozin as Add‐On to Metformin in Patients With Type 2 Diabetes: A 24‐Week, Randomized, Double‐Blind, Placebo‐Controlled Trial,” Diabetes Care 37, no. 6 (2014): 1650–1659. 24722494 10.2337/dc13-2105

[31] S. E. Inzucchi , M. J. Davies , K. Khunti , et al., “Empagliflozin Treatment Effects Across Categories of Baseline HbA1c, Body Weight and Blood Pressure as an Add‐On to Metformin in Patients With Type 2 Diabetes,” Diabetes, Obesity & Metabolism 23, no. 2 (2021): 425–433. 10.1111/dom.14234 PMC7839733 33084149

[32] A. Ishtiaque , S. M. Khan , S. Azhar , M. Mehmood , and S. Shahnawaz , “A Comparison of the Efficacy of Dapagliflozin Metformin Versus Sitagliptin Metformin: In Newly Diagnosed Type 2 Diabetes,” Pakistan Journal of Medical & Health Sciences 16, no. 10 (2022): 459–461.

[33] A. Khan , I. A. Khan , H. Abidi , and M. Ahmed , “Comparison of Empagliflozin and Vildagliptin for Efficacy and Safety in Type 2 Diabetes Mellitus in the Pakistani Population,” Front Endocrinol (Lausanne) 13 (2022): 926633. 36060955 10.3389/fendo.2022.926633 PMC9428695

[34] M. Kitazawa , T. Katagiri , H. Suzuki , et al., “A 52‐Week Randomized Controlled Trial of Ipragliflozin or Sitagliptin in Type 2 Diabetes Combined With Metformin: The N‐ISM Study,” Diabetes, Obesity & Metabolism 23, no. 3 (2021): 811–821. 10.1111/dom.14288 PMC7898334 33416200

[35] M. Kitazawa , M. H. Yamada , M. Iwanaga , et al., “795‐P. Difference in Glycemic Control Between Ipragliflozin (Ipr) and Sitagliptin (Sit) According to Changes in Energy Intake (EI) and Physical Activity (PA): Post‐Hoc Analysis of the NISM Study,” Diabetes 70 (2021): 795‐P.

[36] C. H. Lu , K. W. Min , L. M. Chuang , S. Kokubo , S. Yoshida , and B. S. Cha , “Efficacy, Safety, and Tolerability of Ipragliflozin in Asian Patients With Type 2 Diabetes Mellitus and Inadequate Glycemic Control With Metformin: Results of a Phase 3 Randomized, Placebo‐Controlled, Double‐Blind, Multicenter Trial,” J Diabetes Investig 7, no. 3 (2016): 366–373. 10.1111/jdi.12422 PMC4847891 27330723

[37] K. W. Min , B. J. Ku , J. H. Lee , et al., “Addition of Ipragliflozin to Metformin Treatment in Korean Patients With Type 2 Diabetes Mellitus: Subgroup Analysis of a Phase 3 Trial,” Diabetes & Metabolism Journal 41, no. 2 (2017): 135–145. 28447440 10.4093/dmj.2017.41.2.135 PMC5409005

[38] H. W. Rodbard , J. Rosenstock , L. H. Canani , et al., “Oral Semaglutide Versus Empagliflozin in Patients With Type 2 Diabetes Uncontrolled on Metformin: The PIONEER 2 Trial,” Diabetes Care 42, no. 12 (2019): 2272–2281. 31530666 10.2337/dc19-0883

[39] E. Montanya , M. T. Abildlund , E. B. Kreiner , O. Mosenzon , S. Rosenlund , and T. Vilsboll , “25. Glycaemic Variability of Oral Semaglutide vs Empagliflozin: A Post‐Hoc Analysis of PIONEER 2,” Diabetologia 64, no. 1 (2021): S257.

[40] D. Shabeer , E. Montanya , M. T. Abildlund , et al., “25. Glycemic Variability of Oral Semaglutide Versus Empagliflozin: A Post Hoc Analysis of PIONEER 2,” Indian Journal of Endocrinology and Metabolism 26, no. 1 (2022): S14–S15.

[41] F. K. Knop , B. Cariou , E. Christiansen , et al., “34. Time Spent in Glycaemic Control After Initiating Treatment With Oral Semaglutide vs Empagliflozin: An Exploratory Analysis of the PIONEER 2 Trial,” Diabetologia 64, no. 1 (2021): S249.

[42] V. R. Aroda , J. Eliasson , B. Malling , et al., “609. Multifactorial Risk Reduction With Oral Semaglutide vs Comparators in the Treatment of Type 2 Diabetes,” Diabetologia 65, no. Suppl 1 (2022): S312.

[43] J. Rosenstock , B. Cariou , E. Christiansen , et al., “670‐P. Time Spent in Glycemic Control After Initiating Treatment With Oral Semaglutide vs. Empagliflozin: An Exploratory Analysis of the PIONEER 2 Trial,” Diabetes 70 (2021): 670‐P.

[44] Y. Tang , Q. Sun , X. Y. Bai , Y. F. Zhou , Q. L. Zhou , and M. Zhang , “Effect of Dapagliflozin on Obstructive Sleep Apnea in Patients With Type 2 Diabetes: A Preliminary Study,” Nutrition & Diabetes 9, no. 1 (2019): 32p. 31685792 10.1038/s41387-019-0098-5 PMC6828696

[45] L. Ji , Y. Liu , H. Miao , et al., “Safety and Efficacy of Ertugliflozin in Asian Patients With Type 2 Diabetes Mellitus Inadequately Controlled With Metformin Monotherapy: VERTIS Asia,” Diabetes, Obesity & Metabolism 21, no. 6 (2019): 1474–1482. 10.1111/dom.13681 PMC7379575 30830724

[46] W. T. Cefalu , L. A. Leiter , K. H. Yoon , et al., “Efficacy and Safety of Canagliflozin Versus Glimepiride in Patients With Type 2 Diabetes Inadequately Controlled With Metformin (CANTATA‐SU): 52 Week Results From a Randomised, Double‐Blind, Phase 3 Non‐Inferiority Trial,” Lancet 382, no. 9896 (2013): 941–950. 23850055 10.1016/S0140-6736(13)60683-2

[47] L. A. Leiter , G. Langslet , U. Vijapurkar , M. J. Davies , and W. Canovatchel , “Simultaneous Reduction in Both HbA1c and Body Weight With Canagliflozin Versus Glimepiride in Patients With Type 2 Diabetes on Metformin,” Diabetes Therapy 7, no. 2 (2016): 269–278. 26984361 10.1007/s13300-016-0163-1 PMC4900973

[48] C. A. Patel , R. A. Bailey , U. Vijapurkar , G. Meininger , and L. Blonde , “A Post‐Hoc Analysis of the Comparative Efficacy of Canagliflozin and Glimepiride in the Attainment of Type 2 Diabetes‐Related Quality Measures,” BMC Health Services Research 16 (2016): 356. 27495291 10.1186/s12913-016-1607-z PMC4974722

[49] H. J. Heerspink , M. Desai , M. Jardine , D. Balis , G. Meininger , and V. Perkovic , “Canagliflozin Slows Progression of Renal Function Decline Independently of Glycemic Effects,” Journal of the American Society of Nephrology: JASN 28, no. 1 (2017): 368–375. 27539604 10.1681/ASN.2016030278 PMC5198289

[50] J. Rosenstock , L. Chuck , M. González‐Ortiz , et al., “Initial Combination Therapy With Canagliflozin Plus Metformin Versus Each Component as Monotherapy for Drug‐Naïve Type 2 Diabetes,” Diabetes Care 39, no. 3 (2016): 353–362. 26786577 10.2337/dc15-1736

[51] M. V. Shestakova , J. P. H. Wilding , W. Wilpshaar , R. Tretter , V. L. Orlova , and A. F. Verbovoy , “A Phase 3 Randomized Placebo‐Controlled Trial to Assess the Efficacy and Safety of Ipragliflozin as an Add‐On Therapy to Metformin in Russian Patients With Inadequately Controlled Type 2 Diabetes Mellitus,” Diabetes Research and Clinical Practice 146 (2018): 240–250. 30391333 10.1016/j.diabres.2018.10.018

[52] W. Yang , P. Han , K. W. Min , et al., “Efficacy and Safety of Dapagliflozin in Asian Patients With Type 2 Diabetes After Metformin Failure: A Randomized Controlled Trial,” Journal of Diabetes 8, no. 6 (2016): 796–808. 26589253 10.1111/1753-0407.12357

[53] R. R. Henry , A. V. Murray , M. H. Marmolejo , D. Hennicken , A. Ptaszynska , and J. F. List , “Dapagliflozin, Metformin XR, or Both: Initial Pharmacotherapy for Type 2 Diabetes, a Randomised Controlled Trial,” International Journal of Clinical Practice 66, no. 5 (2012): 446–456. 22413962 10.1111/j.1742-1241.2012.02911.x

[54] F. Lavalle‐González , A. Januszewicz , J. Davidson , et al., “Efficacy and Safety of Canagliflozin Compared With Placebo and Sitagliptin in Patients With Type 2 Diabetes on Background Metformin Monotherapy: A Randomised Trial,” Diabetologia 56 (2013): 2582–2592. 24026211 10.1007/s00125-013-3039-1 PMC3825495

[55] C. J. Bailey , J. L. Gross , A. Pieters , A. Bastien , and J. F. List , “Effect of Dapagliflozin in Patients With Type 2 Diabetes Who Have Inadequate Glycaemic Control With Metformin: A Randomised, Double‐Blind, Placebo‐Controlled Trial,” Lancet 375, no. 9733 (2010): 2223–2233. 20609968 10.1016/S0140-6736(10)60407-2

[56] J. Bolinder , Ö. Ljunggren , J. Kullberg , et al., “Effects of Dapagliflozin on Body Weight, Total Fat Mass, and Regional Adipose Tissue Distribution in Patients With Type 2 Diabetes Mellitus With Inadequate Glycemic Control on Metformin,” Journal of Clinical Endocrinology and Metabolism 97, no. 3 (2012): 1020–1031. 22238392 10.1210/jc.2011-2260

[57] A. Kashiwagi , K. Kazuta , K. Goto , S. Yoshida , E. Ueyama , and A. Utsuno , “Ipragliflozin in Combination With Metformin for the Treatment of Japanese Patients With Type 2 Diabetes: ILLUMINATE, a Randomized, Double‐Blind, Placebo‐Controlled Study,” Diabetes, Obesity & Metabolism 17, no. 3 (2015): 304–308. 10.1111/dom.12331 PMC4342773 24919820

[58] M. A. Nauck , S. Del Prato , J. J. Meier , et al., “Dapagliflozin Versus Glipizide as Add‐On Therapy in Patients With Type 2 Diabetes Who Have Inadequate Glycemic Control With Metformin: A Randomized, 52‐Week, Double‐Blind, Active‐Controlled Noninferiority Trial,” Diabetes Care 34, no. 9 (2011): 2015–2022. 21816980 10.2337/dc11-0606 PMC3161265

[59] R. A. DeFronzo , A. Lewin , S. Patel , et al., “Combination of Empagliflozin and Linagliptin as Second‐Line Therapy in Subjects With Type 2 Diabetes Inadequately Controlled on Metformin,” Diabetes Care 38, no. 3 (2015): 384–393. 25583754 10.2337/dc14-2364

[60] S. Efstathiou , I. Skeva , and T. Mountokalakis , “Empagliflozin May Attenuate Adipose Tissue Inflammation and Arterial Stiffness in Normotensive Type 2 Diabetics,” Journal of Hypertension 36 (2018): e84.

[61] J. Li , Y. Gong , C. Li , Y. Lu , Y. Liu , and Y. Shao , “Long‐Term Efficacy and Safety of Sodium‐Glucose Cotransporter‐2 Inhibitors as Add‐On to Metformin Treatment in the Management of Type 2 Diabetes Mellitus: A Meta‐Analysis,” Medicine 96, no. 27 (2017): e7201. 28682870 10.1097/MD.0000000000007201 PMC5502143

[62] Y. Ingrasciotta , M. P. Bertuccio , S. Crisafulli , et al., “Real World Use of Antidiabetic Drugs in the Years 2011–2017: A Population‐Based Study From Southern Italy,” International Journal of Environmental Research and Public Health 17, no. 24 (2020): 9514. 33353081 10.3390/ijerph17249514 PMC7765957

[63] Y. S. Lyu , S. Oh , J. H. Kim , S. Y. Kim , and M. H. Jeong , “Comparison of SGLT2 Inhibitors With DPP‐4 Inhibitors Combined With Metformin in Patients With Acute Myocardial Infarction and Diabetes Mellitus,” Cardiovascular Diabetology 22, no. 1 (2023): 185. 37481509 10.1186/s12933-023-01914-4 PMC10362625

[64] B. Ahrén and J. E. Foley , “Improved Glucose Regulation in Type 2 Diabetic Patients With DPP‐4 Inhibitors: Focus on Alpha and Beta Cell Function and Lipid Metabolism,” Diabetologia 59, no. 5 (2016): 907–917. 26894277 10.1007/s00125-016-3899-2

[65] H. Ma , Y. H. Lin , L. Z. Dai , C. S. Lin , Y. Huang , and S. Y. Liu , “Efficacy and Safety of GLP‐1 Receptor Agonists Versus SGLT‐2 Inhibitors in Overweight/Obese Patients With or Without Diabetes Mellitus: A Systematic Review and Network Meta‐Analysis,” BMJ Open 13, no. 3 (2023): e061807. 10.1136/bmjopen-2022-061807 PMC10008474 36882248

[66] J. F. List , V. Woo , E. Morales , W. Tang , and F. T. Fiedorek , “Sodium‐Glucose Cotransport Inhibition With Dapagliflozin in Type 2 Diabetes,” Diabetes Care 32, no. 4 (2009): 650–657. 19114612 10.2337/dc08-1863 PMC2660449

[67] R. A. DeFronzo , J. B. Buse , T. Kim , et al., “Once‐Daily Delayed‐Release Metformin Lowers Plasma Glucose and Enhances Fasting and Postprandial GLP‐1 and PYY: Results From Two Randomised Trials,” Diabetologia 59, no. 8 (2016): 1645–1654. 27216492 10.1007/s00125-016-3992-6 PMC4930485

[68] A. Napolitano , S. Miller , A. W. Nicholls , et al., “Novel Gut‐Based Pharmacology of Metformin in Patients With Type 2 Diabetes Mellitus,” PLoS One 9, no. 7 (2014): e100778. 24988476 10.1371/journal.pone.0100778 PMC4079657

[69] N. Molugulu , L. S. Yee , Y. T. Ye , et al., “Systematic Review of Metformin Monotherapy and Dual Therapy With Sodium Glucose Co‐Transporter 2 Inhibitor (SGLT‐2) in Treatment of Type 2 Diabetes Mellitus,” Diabetes Research and Clinical Practice 132 (2017): 157–168. 28797524 10.1016/j.diabres.2017.07.025

[70] W. Ghusn , M. D. Hurtado , and A. Acosta , “Weight‐Centric Treatment of Type 2 Diabetes Mellitus,” Obes Pillars 4 (2022): 100045. 37990663 10.1016/j.obpill.2022.100045 PMC10662009

[71] H. Sharma , “Statistical Significance or Clinical Significance? A Researcher's Dilemma for Appropriate Interpretation of Research Results,” Saudi Journal of Anaesthesia 15, no. 4 (2021): 431–434. 34658732 10.4103/sja.sja_158_21 PMC8477766

[72] M. Dankers , M. H. Nelissen‐Vrancken , B. H. Hart , A. C. Lambooij , L. van Dijk , and A. K. Mantel‐Teeuwisse , “Alignment Between Outcomes and Minimal Clinically Important Differences in the Dutch Type 2 Diabetes Mellitus Guideline and Healthcare Professionals' Preferences,” Pharmacology Research & Perspectives 9, no. 3 (2021): e00750. 33934550 10.1002/prp2.750 PMC8244004

[73] B. Lyu , Y. J. Hwang , E. Selvin , et al., “Glucose‐Lowering Agents and the Risk of Hypoglycemia: A Real‐World Study,” Journal of General Internal Medicine 38, no. 1 (2023): 107–114. 35831767 10.1007/s11606-022-07726-8 PMC9849518

[74] J. Z. Zhao , E. D. Weinhandl , A. M. Carlson , and W. L. S. Peter , “Hypoglycemia Risk With SGLT2 Inhibitors or Glucagon‐Like Peptide 1 Receptor Agonists Versus Sulfonylureas Among Medicare Insured Adults With CKD in the United States,” Kidney Medicine 4, no. 8 (2022): 100510. 35898692 10.1016/j.xkme.2022.100510 PMC9310119

[75] E. D'Andrea , D. J. Wexler , S. C. Kim , J. M. Paik , E. Alt , and E. Patorno , “Comparing Effectiveness and Safety of SGLT2 Inhibitors vs DPP‐4 Inhibitors in Patients With Type 2 Diabetes and Varying Baseline HbA1c Levels,” JAMA Internal Medicine 183, no. 3 (2023): 242–254. 36745425 10.1001/jamainternmed.2022.6664 PMC9989905

[76] W. Alkabbani , A. Zongo , J. K. Minhas‐Sandhu , et al., “Sodium‐Glucose Cotransporter‐2 Inhibitors and Urinary Tract Infections: A Propensity Score–Matched Population‐Based Cohort Study,” Canadian Journal of Diabetes 46, no. 4 (2022): 392–403.e313. 35513988 10.1016/j.jcjd.2021.12.005

[77] J. J. Gorgojo‐Martínez , J. L. Górriz , A. Cebrián‐Cuenca , A. Castro Conde , and M. Velasco Arribas , “Clinical Recommendations for Managing Genitourinary Adverse Effects in Patients Treated With SGLT‐2 Inhibitors: A Multidisciplinary Expert Consensus,” Journal of Clinical Medicine 13, no. 21 (2024): 6509. 39518647 10.3390/jcm13216509 PMC11546491

[78] J. A. Sterne , A. J. Sutton , J. P. Ioannidis , et al., “Recommendations for Examining and Interpreting Funnel Plot Asymmetry in Meta‐Analyses of Randomised Controlled Trials,” BMJ 343 (2011): d4002. 21784880 10.1136/bmj.d4002

[79] O. Efthimiou , “Practical Guide to the Meta‐Analysis of Rare Events,” Evidence‐Based Mental Health 21, no. 2 (2018): 72–76. 29650528 10.1136/eb-2018-102911 PMC10270432

