# Supplementary material for: Comparing SGLT2i and Other Oral Antidiabetic Drugs as Dual Therapy Add‐On to Metformin in Type 2 Diabetes: A Systematic Review and Meta‐Analysis
Source: Endocrinol Diabetes Metab. 2026 Feb 12;9(2):e70176. doi: 10.1002/edm2.70176 (PMC12895371; doi:10.1002/edm2.70176)
Supplement: Supplementary file 1 — Table S1: Search strategies. Table S2: PICOS eligibility criteria for SLR update. Table S3: Baseline patient characteristics. Table S4: Risk of bias. Figure S1: Funnel plots for main analyses. Figure S2: Funnel plots for subgroup analyses comparing (a) DPP‐4i plus metformin and (b) SU plus metformin Figure S3: Results from efficacy analyses of SGLT‐2i plus metformin for (a) change in HbA1c at Week 52 in subgroup analysis against SU plus metformin, (b) change in HbA1c at Week 24 in subgroup analysis against DPP‐4i plus metformin, (c) change in HbA1c at Week 52 in subgroup analysis against DPP‐4i plus metformin, (d) HbA1c level at Week 24 in main analysis, (e) HbA1c level at Week 24 in subgroup analysis comparing against DPP‐4i plus metformin and (f) treatment failure at Week 52 in main analysis. Figure S4: Results from safety analyses of SGLT‐2i plus metformin at Week 52 for (a) UTI in subgroup analysis comparing against DPP‐4i plus metformin and (b) genital infection in main analysis. Figure S5: Results from safety analyses of SGLT‐2i plus metformin for overall serious AEs at Week 52 in (a) main analysis and (b) subgroup analysis comparing against DPP‐4i plus metformin. Figure S6: Results from main safety analyses of SGLT‐2i plus metformin at Week 52 for (a) all‐cause mortality, (b) stroke and (c) diarrhoea. [file EDM2-9-e70176-s001.docx]

Table S1 Search strategies

| Electronic Databases  EBM Reviews - Cochrane Central Register of Controlled Trials April 2023, EBM Reviews - Cochrane Database of Systematic Reviews 2005 to May 23, 2023, Embase 1974 to May 22, 2023, Ovid MEDLINE(R) and Epub Ahead of Print, In-Process, In-Data-Review & Other Non-Indexed Citations and Daily 1946 to May 22, 2023 | | |
| --- | --- | --- |
| Term Group | **#** | **Search Terms** |
| T2DM |  | exp Diabetes Mellitus, Type 2/ or "Diabetes Mellitus, Type 2".kw. |
|  |  | Diabetes Mellitus Type 2.mp. |
|  |  | Diabetes Mellitus Type II.mp. |
|  |  | DM type 2.mp. |
|  |  | DM type II.mp. |
|  |  | Type 2 Diabetes.mp. |
|  |  | Type II Diabetes.mp. |
|  |  | Diabetes Type II.mp. |
|  |  | Diabetes Type 2.mp |
|  |  | T2DM.mp. |
|  |  | DMT2.mp. |
|  |  | Or/1-11 |
| Metformin |  | metformin.mp. |
|  |  | glucophage.mp. |
|  |  | dimethylbiguanidine.mp. |
|  |  | dimethylguanylguanidine.mp. |
|  |  | exp Metformin/ or Metformin.kw. |
|  |  | (Fortamet or Glucophage or Glucophage XR or Glumetza or Riomet).mp. |
|  |  | Or/13-18 |
| SGLT2i |  | sodium glucose cotransporter 2 inhibitor/ or sodium glucose cotransporter 2/ or sodium-glucose transporter 2/ or sodium glucose transporter 2/ or (sodium glucose cotransporter 2 inhibitor or sodium glucose cotransporter 2 or sodium-glucose transporter 2 or sodium glucose transporter 2).kw. |
|  |  | (((SGLT2 or SGLT 2) adj2 inhibit$) or SGLT2i or SGLT 2i).tw. |
|  |  | Flozin$.mp. |
|  |  | (dapagliflozin or Forxiga$ or Farxiga$ or Andatang$ or oxra$). ti,ab,kw,kf. |
|  |  | (canagliflozin or Invokana$ or Canaglu$ or Sulisent$). ti,ab,kw,kf. |
|  |  | (empagliflozin or Jardiance$ or Oboravo$).mp. |
|  |  | (Ertugliflozin or Steglatro$).mp. |
|  |  | (Sotagliflozin or Zynquista$).mp. |
|  |  | (Remogliflozin or remozen$ or zucator$).mp. |
|  |  | (Tofogliflozin or Apleway$ or Deberza$).mp. |
|  |  | (Ipragliflozin or suglat$).mp. |
|  |  | atigliflozin.mp. |
|  |  | bexagliflozin.mp. |
|  |  | enavogliflozin.mp. |
|  |  | licogliflozin.mp. |
|  |  | (luseogliflozin or Lusefi$).mp. |
|  |  | mizagliflozin.mp. |
|  |  | sergliflozin.mp. |
|  |  | Or/20-37 |
| Combination therapies |  | exp *drug combinations/ or "*drug combinations".kw. |
|  |  | (fixed dose or fixeddose or multidose or multi dose).mp. |
|  |  | (polypill$ or policap$).mp. |
|  |  | ((drug or pill or loose dose) adj combin$).mp. |
|  |  | ((multi$ or several) adj2 (ingredient$ or component$)).mp. |
|  |  | (FDC or TPC).mp. |
|  |  | (dual or two drug) adj2 (regimen or combin$ or therapy).mp |
|  |  | Or/39-45 |
| RCTs |  | Randomized Controlled Trials as Topic/ or "randomized controlled trial (topic)"/ |
|  |  | Randomized Controlled Trial/ |
|  |  | Random Allocation/ or randomization/ |
|  |  | Double-Blind Method/ or double blind procedure/ |
|  |  | Single-Blind Method/ or single blind procedure/ |
|  |  | crossover procedure/ |
|  |  | placebo/ or Placebos.ti,ab,kw,kf. |
|  |  | exp Clinical Trials as Topic/ or exp "clinical trial (topic)"/ |
|  |  | Clinical Trial/ |
|  |  | Clinical Trial, Phase I/ or phase 1 clinical trial/ or Clinical Trial, Phase II/ or phase 2 clinical trial/ or Clinical Trial, Phase III/ or phase 3 clinical trial/ or Clinical Trial, Phase IV/ or phase 4 clinical trial/ |
|  |  | Controlled Clinical Trial/ or Adaptive Clinical Trial/ or multicenter study/ |
|  |  | randomized controlled trial.pt. |
|  |  | clinical trial.pt. |
|  |  | (clinical trial, phase i or clinical trial, phase ii or clinical trial, phase iii or clinical trial, phase iv).pt. |
|  |  | (controlled clinical trial or multicenter study).pt. |
|  |  | (clinical adj trial$).ti,ab,kw,kf. |
|  |  | ((singl$ or doubl$ or treb$ or tripl$) adj (blind$3 or mask$3)).ti,ab,kw,kf. |
|  |  | placebo$.ti,ab,kw,kf. |
|  |  | (allocat$ adj2 random$).ti,ab,kw,kf. |
|  |  | Randomi?ed adj2 trial$.ti,ab,kw,kf. |
|  |  | rct.ti,ab,kw,kf. |
|  |  | (Trial or study).ti. |
|  |  | (comparative adj2 (stud* or trial$)).ti,ab,kw,kf. |
|  |  | Or/47-69 |
| Exclusion |  | ("conference abstract" or "conference review").pt. |
|  |  | limit 71 to yr="1946-2020" |
|  |  | exp animals/ not exp humans/ |
|  |  | (comment or editorial or case reports or historical article).pt. |
|  |  | (case stud$ or case report$).ti. |
|  |  | historical article/ |
|  |  | or/72-76 |
| Combination |  | 19 and 38 |
|  |  | 12 and 46 and 78 and 70 |
|  |  | 79 not 77 |
|  |  | Limit 80 to yr="2016-current" |
|  |  | Remove duplicates from 81 |
|  |  | 12 and 46 and 78 |
|  |  | 83 use cctz |
|  |  | 83 use coch |
|  |  | 82 or 84 or 85 |
|  |  | Limit 86 to yr="2016-current" |
|  |  | Remove duplicates from 87 |
| Conference proceedings from 2021, 2022 and 2023 | | |
| - ADA - EASD | | |

Abbreviations: ADA, American Diabetes Association; EASD, European Association for the Study of Diabetes; RCT, randomised controlled trial; SGLT-2i, sodium-glucose cotransporter-2 inhibitors; T2DM, type 2 diabetes mellitus.

Table S2 PICOS eligibility criteria for SLR update

| **Category** | **Inclusion criteria** | **Exclusion criteria** |
| --- | --- | --- |
| **Population** | - Adult patients with T2DM - Populations fulfilling any one of the below criteria were considered as subgroups of interest (but studies were not excluded on the basis of not reporting these subgroups): - Treatment naïve patients with HbA_1c_ >7.5% and/or cardiorenal high risk - Patients with any monotherapy failure who changed to the dual-therapy regimen of metformin with SGLT-2i | - Any other disease - Children or adolescents (<18 years old) |
| **Intervention** | - Dual therapy regimens with metformin and SGLT-2i (co-administered dual therapy or FDC) such as: - Canagliflozin - Dapagliflozin - Empagliflozin - Ertugliflozin - Sotagliflozin - Remogliflozin - Tofogliflozin - Ipragliflozin - Atigliflozin - Bexagliflozin - Enavogliflozin - Licogliflozin - Luseogliflozin - Mizagliflozin - Sergliflozin | - Any other regimen |
| **Comparator** | - Any other oral metformin-containing dual-therapy regimens (co-administered dual therapy or FDC) | - Monotherapy regimens - Dual-therapy regimens without metformin (co-administered dual therapy or FDC) - Dual-therapy regimens assessing different doses of the same SGLT-2i - Triple therapy regimens - Any regimen containing injectable therapy such as insulin or GLP-1 |
| **Outcomes** | - Efficacy outcomes: - HbA_1c_ levels - Treatment failure (lack of efficacy or need for rescue treatment) - Safety outcomes: - Cardiorenal vascular outcomes: - Cardiovascular mortality - All-cause mortality - MI - Stroke - Heart failure - Renal hard endpoints:   - Sustained decline in the eGFR of at least 50%   - End-stage kidney disease   - Death from renal or cardiovascular causes - Serious AEs - Hypoglycaemia - Body weight - Gastrointestinal AEs of any grade - UTI - Genital infection | - Any other outcomes |
| **Study design** | - Parallel-arm RCTs in which treatment was given for 24 weeks or longer   *SLRs and (N)MAs of RCTs considered relevant at the title/abstract review stage were hand-searched for relevant studies. They were excluded during the full-text review stage unless presenting original research themselves* | - Any other study designs |
| **Publication type** | - Original peer-reviewed research - Letters reporting original research - Conference abstracts published in or since 2021 - Studies published since Palmer *et al.* 2016 ran their searches in March 2016 (to avoid duplication of any 2016 studies identified by Palmer *et al.* 2016) | - Any other publication type, including studies not reporting any original research and non-peer-reviewed (e.g., narrative reviews, case studies) - Conference abstracts published prior to 2021 - Studies published prior to Palmer *et al.* searches in March 2016 (to avoid duplication of any 2016 studies identified by Palmer *et al.* 2016) |
| **Other considerations** | - Any location - Human subjects - English language (abstract or full-text articles) | - Animal subjects - In-vitro/preclinical studies - Not reported in the English language |

Abbreviations: AE, adverse event; eGFR, estimated glomerular filtration rate; FDC, fixed-dose combination; GLP-1, glucagon-like peptide-1; HbA_1c_, glycated haemoglobin A1c; (N)MA, (network) meta-analysis; MI, myocardial infarction; PICO, population, intervention, comparator, outcome; RCT, randomised controlled trial; SGLT-2i, sodium-glucose cotransporter-2 inhibitors; SLR: systematic literature review; T2DM, type 2 diabetes mellitus; UTI, urinary tract infection.

Table S3 Baseline patient characteristics

| Study name | Mean age (years) | Male sex (%) | Mean diagnosis duration (years) | Mean treatment duration (months) | Mean HbA_1c_ (%) | Mean weight (kg) |
| --- | --- | --- | --- | --- | --- | --- |
| Gao 2023 | 54.84 | 65.68 | 5.68 | NR | 8.36 | 70.73 |
| Halvorsen 2023 | 59.6 | 58.22 | 8.66 | NR | 8.01 | 89.09 |
| Ishtiaque 2022 | 47.21 | 50.56 | NR | NR | 7.64 | NR |
| Khan 2022 | 50.83 | 45.79 | NR | NR | 9.32 | NR |
| Tang 2019 | 56.99 | 60 | NR | NR | 8.42 | NR |
| N-ISM | 59.18 | 61.26 | 9.74 | NR | 7.53 | 70.93 |
| Lu 2016 | 53.66 | 45.29 | 6.16 | NR | 7.74 | 70.40 |
| VERTIS Asia | 56.43 | 55.53 | 6.97 | NR | 8.1 | 70.34 |
| PIONEER 2 | 57.50 | 50.55 | 7.45 | NR | 8.1 | 91.60 |
| Rosenstock 2016 | 54.93 | 47.68 | 3.17 | NR | 8.83 | 90.6 |
| IMPRESSION | 58.32 | 46.28 | 6.56 | NR | 8.36 | 93.45 |
| Yang 2016 | 53.74 | 54.28 | 4.94 | NR | 8.13 | 71.04 |
| Henry 2012 | 51.80 | 46.18 | 1.83 | 5.5 | 9.15 | 86.38 |
| CANTATA-D | 55.41 | 47.14 | 6.86 | 6 | 7.91 | 87.20 |
| Bailey 2010 | 53.93 | 53.24 | 6.07 | 5.5 | 8.05 | 85.90 |
| Bolinder 2012 | 60.70 | 55.56 | 5.75 | 5.52 | 7.17 | 91.49 |
| EMPA-REG MET | 55.70 | 56.68 | NR | 5.5 | 7.89 | 81.18 |
| ILLUMINATE | 56.7 | 58.9 | 7.68 | 5.5 | 8.29 | 68.18 |
| CANTATA-SU | 55.57 | 52.09 | 6.60 | 12 | 7.8 | 86.67 |
| EMPA-REG H2H-SU | 55.95 | 54.99 | NR | 24 | 7.92 | 82.75 |
| Nauck 2011 | 58.50 | 55.10 | 6.50 | 12 | 7.7 | NR |
| DeFronzo 2015 | 55.92 | 51.09 | NR | 12 | 8.01 | 86.31 |
| Efstathiou 2015 | NR | NR | 6.5 | 5.5 | NR | NR |

Abbreviations: HbA_1c_, glycated haemoglobin A1c; NR, not reported.

Table S4 Risk of bias

| **Study name** | **Treatment comparisons** | **Sequence generation** | **Allocation concealment** | **Masking of participants and investigators** | **Outcome assessment** | **Attrition** | **Selective outcome reporting** | **Sponsor involved in authorship and/or data management** |
| --- | --- | --- | --- | --- | --- | --- | --- | --- |
| Gao 2023 | SGLT-2i vs PBO | Low risk | Low risk | Low risk | Unclear | Low risk | Low risk | Y |
| Halvorsen 2023 | SGLT-2i vs SU | Unclear | Unclear | Low risk | Low risk | Low risk | Low risk | Y |
| Ishtiaque 2022 | SGLT-2i vs DPP-4i | Low risk | Unclear | Unclear | Unclear | High risk | Low risk | N |
| Khan 2022 | SGLT-2i vs DPP-4i | Low risk | Unclear | High risk | High risk | Low risk | Low risk | N |
| Tang 2019 | SGLT-2i vs SU | Low risk | Low risk | Unclear | Unclear | Unclear | Low risk | N |
| N-ISM | SGLT-2i vs DPP-4i | Low risk | Unclear | High risk | High risk | Low risk | Low risk | N |
| Lu 2016 | SGLT-2i vs PBO | Low risk | Low risk | Low risk | Low risk | Low risk | Low risk | Y |
| VERTIS Asia | SGLT-2i vs PBO | Low risk | Unclear | Low risk | Unclear | Low risk | Low risk | Y |
| PIONEER 2 | SGLT-2i vs GLP-1RA | Unclear | Unclear | High risk | High risk | Low risk | Low risk | Y |
| Rosenstock 2016 | SGLT-2i vs PBO | Low risk | Unclear | Low risk | Unclear | Low risk | Low risk | Y |
| IMPRESSION | SGLT-2i vs PBO | Low risk | Unclear | Low risk | Unclear | Low risk | Low risk | Y |
| Yang 2016 | SGLT-2i vs PBO | Low risk | Unclear | Low risk | Low risk | Low risk | Low risk | Y |
| Henry 2012 | SGLT-2i vs PBO | Low risk | Low risk | Low risk | Unclear | High risk | Low risk | Y |
| CANTATA-D | SGLT-2i vs DPP-4i  vs PBO | Low risk | Unclear | Low risk | Unclear | Low risk | Low risk | Y |
| Bailey 2010 | SGLT-2i vs PBO | Low risk | Low risk | Low risk | Unclear | Low risk | Low risk | Y |
| Bolinder 2012 | SGLT-2i vs PBO | Low risk | Unclear | Low risk | Unclear | High risk | Low risk | Y |
| EMPA-REG MET | SGLT-2i vs PBO | Low risk | Low risk | Low risk | Unclear | Low risk | Low risk | Y |
| ILLUMINATE | SGLT-2i vs PBO | Unclear | Unclear | Low risk | Unclear | Low risk | Low risk | Y |
| CANTATA-SU | SGLT-2i vs SU | Low risk | Low risk | Low risk | Unclear | Low risk | Low risk | Y |
| EMPA-REG H2H-SU | SGLT-2i vs SU | Low risk | Low risk | Low risk | Unclear | High risk | Low risk | Y |
| Nauck 2011 | SGLT-2i vs SU | Low risk | Low risk | Low risk | Unclear | High risk | Low risk | Y |
| DeFronzo 2015 | SGLT-2i vs DPP-4i | Low risk | Low risk | Low risk | Unclear | High risk | Low risk | Y |
| Efstathiou 2015 | SGLT-2i vs DPP-4i | Unclear | Unclear | High risk | Unclear | Unclear | High risk | N |

Abbreviations: DPP-4i, dipeptidyl peptidase-4 inhibitors; GLP-1RA, glucagon-like peptide-1 receptor agonist; N, no; PBO, placebo; SGLT-2i, sodium-glucose cotransporter-2 inhibitors; SU, sulphonylurea; Y, yes.


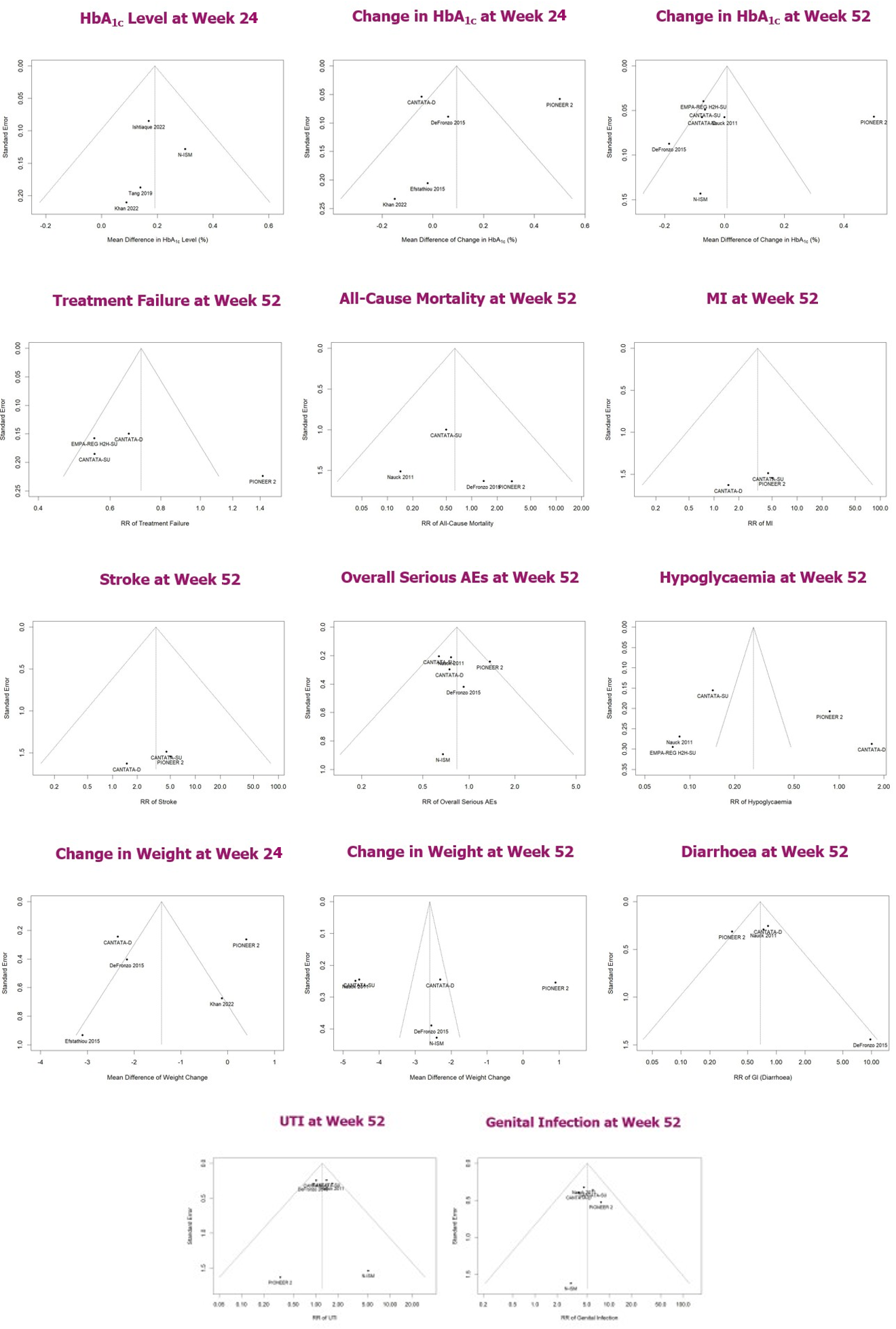


Figure S1 Funnel plots for main analyses

Abbreviation: AE, adverse event; HbA1c, haemoglobin A1c; MI, myocardial infarction; UTI, urinary tract infection.


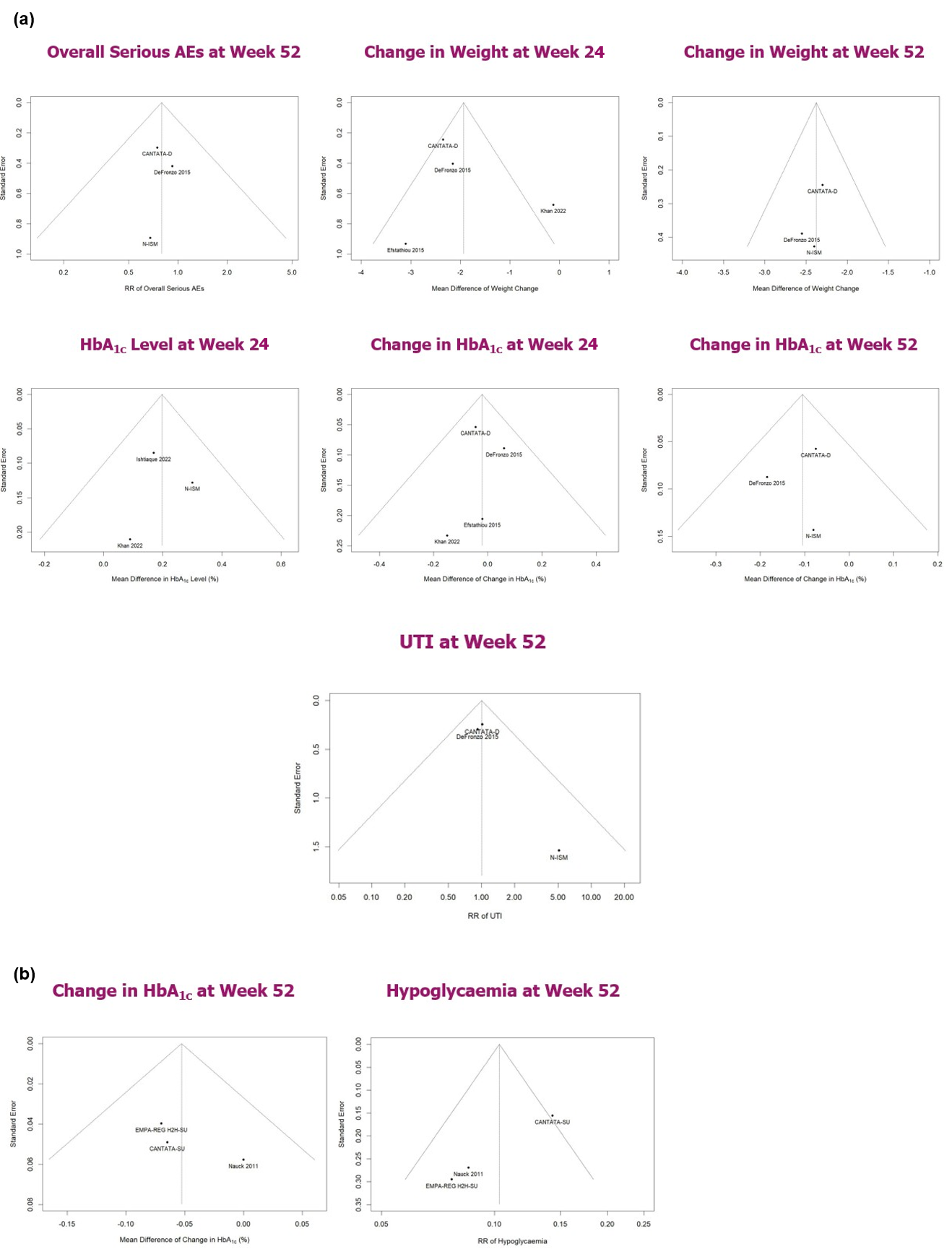


Figure S2 Funnel plots for subgroup analyses comparing (a) DPP-4i plus metformin and (b) SU plus metformin

Abbreviations: AE, adverse event; DPP-4i, dipeptidyl peptidase-4 inhibitors; HbA_1c_, haemoglobin A1c; SU, sulphonylurea; UTI, urinary tract infection.


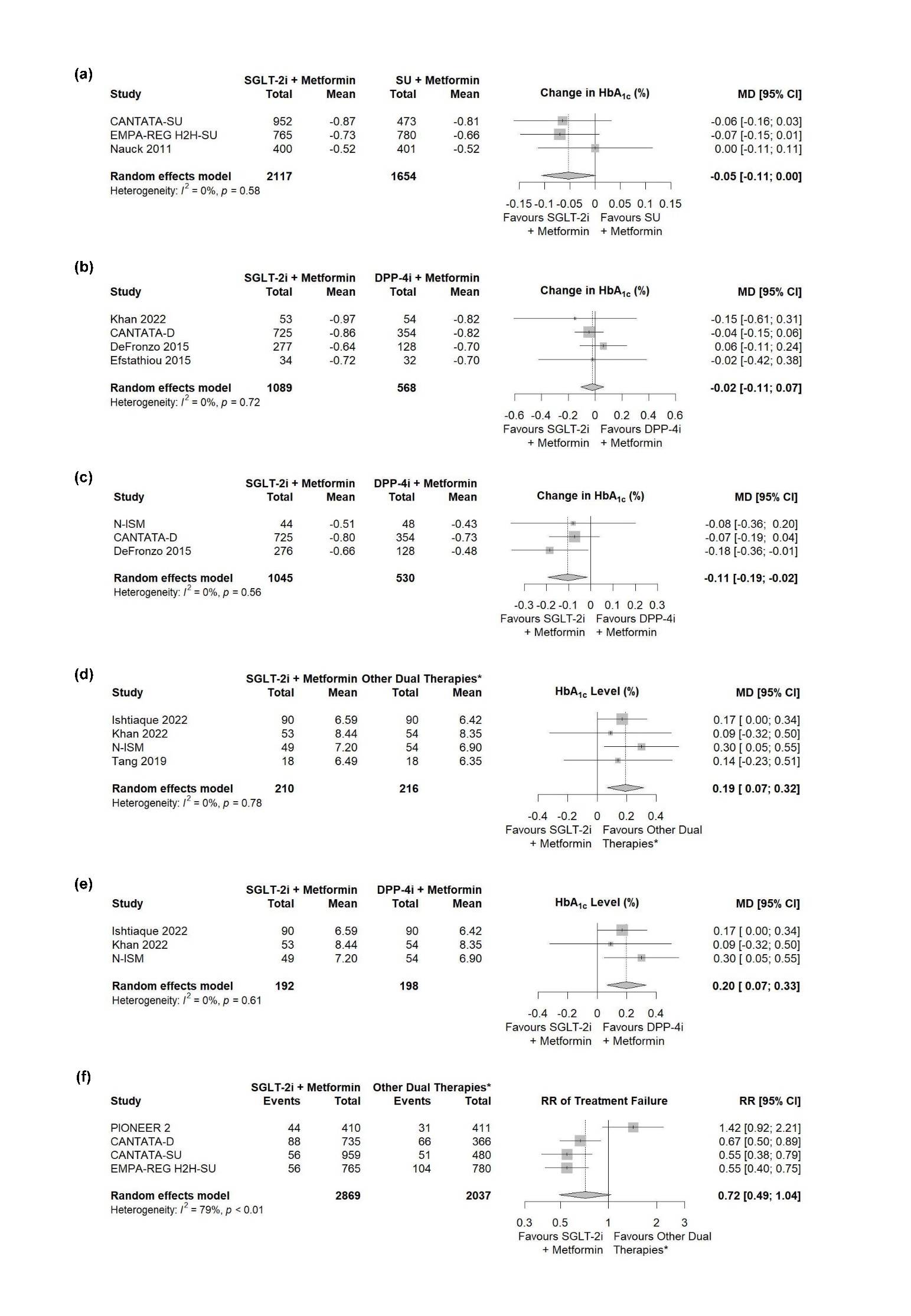


Figure S3 Results from efficacy analyses of SGLT-2i plus metformin for (a) change in HbA_1c_ at Week 52 in subgroup analysis against SU plus metformin, (b) change in HbA_1c_ at Week 24 in subgroup analysis against DPP-4i plus metformin, (c) change in HbA_1c_ at Week 52 in subgroup analysis against DPP-4i plus metformin, (d) HbA_1c_ level at Week 24 in main analysis, (e) HbA_1c_ level at Week 24 in subgroup analysis comparing against DPP-4i plus metformin, and (f) treatment failure at Week 52 in main analysis

^*^Consisting of DPP-4i, GLP-1RA or SU in combination with metformin.

Abbreviations: CI, confidence interval; DPP-4i, dipeptidyl peptidase-4 inhibitors; GLP-1RA, glucagon-like peptide-1 receptor agonist; HbA_1c_, haemoglobin A1c; MD, mean difference; RR, relative risk; SGLT-2i, sodium-glucose cotransporter-2 inhibitors; SU, sulphonylurea.

**
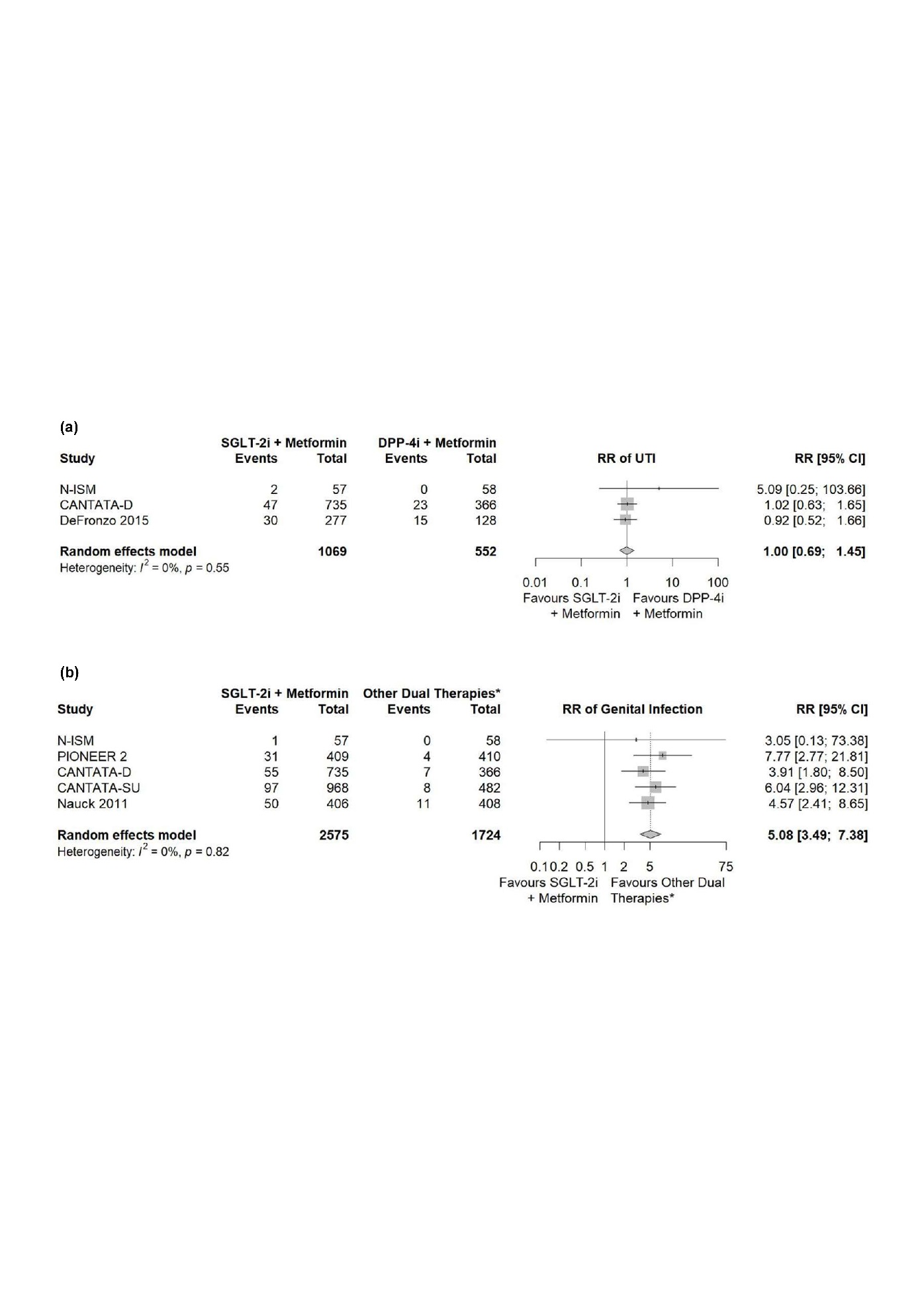
**

Figure S4 Results from safety analyses of SGLT-2i plus metformin at Week 52 for (a) UTI in subgroup analysis comparing against DPP-4i plus metformin and (b) genital infection in main analysis

^*^Consisting of DPP-4i, GLP-1RA or SU in combination with metformin.

Abbreviations: CI, confidence interval; DPP-4i, dipeptidyl peptidase-4 inhibitors; GLP-1RA, glucagon-like peptide-1 receptor agonist; RR, relative risk; SGLT-2i, sodium-glucose cotransporter-2 inhibitors; SU, sulphonylurea; UTI, urinary tract infection.


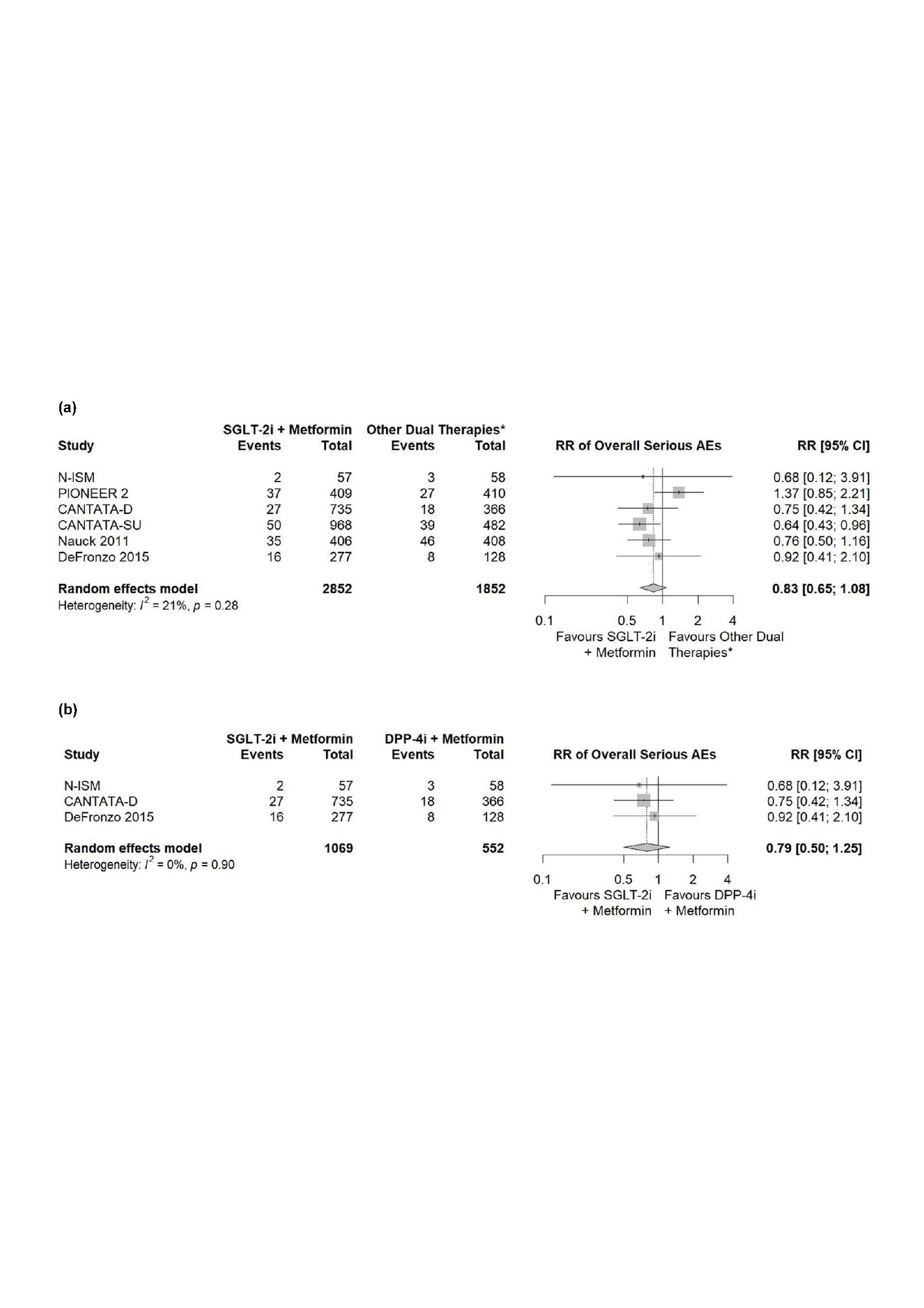


Figure S5 Results from safety analyses of SGLT-2i plus metformin for overall serious AEs at Week 52 in (a) main analysis and (b) subgroup analysis comparing against DPP-4i plus metformin

^*^Consisting of DPP-4i, GLP-1RA or SU in combination with metformin.

Abbreviations: AE, adverse event; CI, confidence interval; DPP-4i, dipeptidyl peptidase-4 inhibitors; GLP-1RA, glucagon-like peptide-1 receptor agonist; RR, relative risk; SGLT-2i, sodium-glucose cotransporter-2 inhibitors; SU, sulphonylurea.


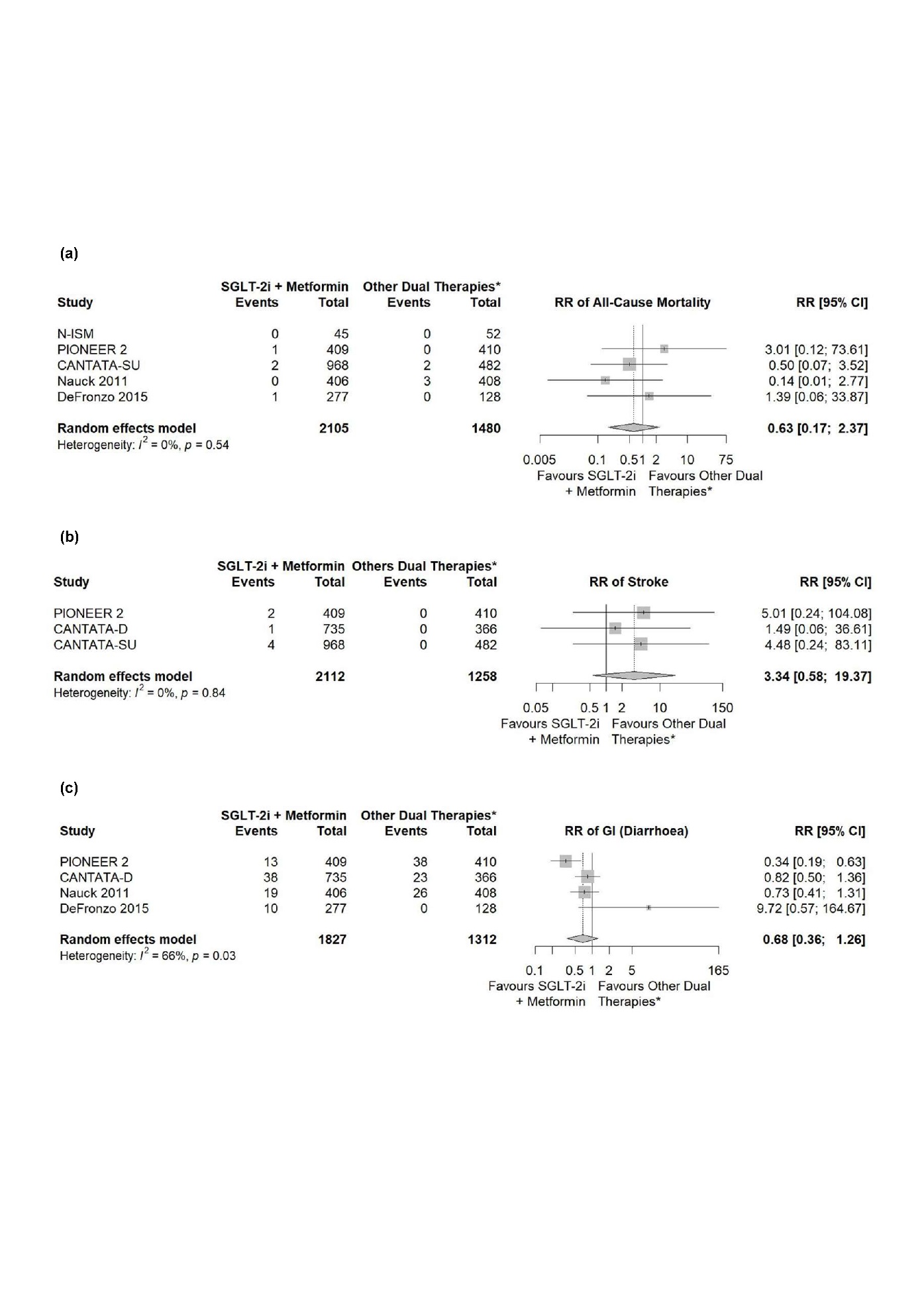


Figure S6 Results from main safety analyses of SGLT-2i plus metformin at Week 52 for (a) all-cause mortality, (b) stroke, and (c) diarrhoea

^*^Consisting of DPP-4i, GLP-1RA or SU in combination with metformin.

Abbreviations: CI, confidence interval; DPP-4i, dipeptidyl peptidase-4 inhibitors; GI, gastrointestinal; GLP-1RA, glucagon-like peptide-1 receptor agonist; RR, relative risk; SGLT-2i, sodium-glucose cotransporter-2 inhibitors; SU, sulphonylurea.
